# Effectiveness of primary school-based interventions in improving oral health of children in low- and middle-income countries: a systematic review and meta-analysis

**DOI:** 10.1186/s12903-022-02291-2

**Published:** 2022-06-29

**Authors:** Peter Akera, Sean E. Kennedy, Raghu Lingam, Mark J. Obwolo, Aletta E. Schutte, Robyn Richmond

**Affiliations:** 1 School of Population Health, Faculty of Medicine and Health, University of New South Wales, Sydney, Australia; 2 School of Women’s and Children’s Health, Faculty of Medicine and Health, University of New South Wales, Sydney, Australia; 3 Department of Public Health, Faculty of Medicine, Gulu University, P.O. Box 166, Gulu, Uganda; 4 The George Institute for Global Health, Sydney, Australia

**Keywords:** Oral health, Primary school, Children, Dental caries, Meta-analysis

## Abstract

**Background:**

Risk factors for oral disease can potentially be ameliorated by school-based interventions. This review evaluates the effectiveness of primary school-based interventions in improving oral health among children in low-and middle-income countries (LMICs).

**Methods:**

Our systematic review was conducted in accordance with the Joanna Briggs Institute methodology for systematic reviews of effectiveness. Medline, Embase, Global Health, CINAHL, Emcare, Scopus, Web of Science, WHO website, Google Advanced and Google Scholar were searched for experimental and observational studies published between 1995 and 2021 in English. Quality assessment and data extraction of the articles were performed by two independent reviewers. The primary outcome was decayed, missing, and filled teeth/surfaces [dmft(s)/DMFT(S)] scores. Seven meta-analyses were conducted.

**Results:**

The search yielded 1178 publications and after removing duplicates, 753 remained. A further 648 publications were excluded after screening titles and abstracts. 105 publications were reviewed in full and 34 were included. Narrative synthesis showed school-based interventions had a positive effect on oral health outcomes. Meta-analysis showed a significant positive effect on dental caries measured by DMFT scores (standardised mean difference (SMD) =  − 0.33; 95% CI − 0.56 to − 0.10; *P* = 0.005), net increment in DMFS scores (SMD =  − 1.09; 95% CI − 1.91 to − 0.27; *P* = 0.009), dmft and DMFT/S score > 1 (Risk Ratio = 0.70; 95% CI 0.53 to 0.94; *P* = 0.02) and plaque scores (SMD =  − 0.32; 95% CI − 0.46 to − 0.18; *P* < 0.00001). Non-significant positive effect was observed for dental caries measured by net increment in DMFT scores (SMD =  − 0.34; 95% CI − 0.69 to 0.02; *P* = 0.06) and DMFS scores (SMD =  − 0.26; 95% CI − 0.70 to 0.18; *P* = 0.24), and gingival health (SMD = 0.12; 95% CI − 0.32 to 0.55; *P* = 0.60). Certainty of evidence was assessed as very low for all oral health outcomes.

**Conclusion:**

School-based interventions can be effective in reducing the burden of oral disease among primary school children in LMICs, with skills-based education, teacher training, provision of access to oral health services and parental engagement emerging as particularly promising. Further research is required to provide evidence of effectiveness of primary school-based interventions to improve oral health.

*Systematic review registration* The title of this review was registered with PROSPERO (registration number: CRD42020202599).

**Supplementary Information:**

The online version contains supplementary material available at 10.1186/s12903-022-02291-2.

## Background

Oral diseases are a prevalent non-communicable disease (NCD) and a major public health issue worldwide [1, 2], with 60–90% of schoolchildren and adults in low-and-middle income countries (LMICs) having dental caries [3]. Lack of awareness of preventive measures, limited access to oral health services, growing consumption of sugars and inadequate exposure to fluorides are all associated with increased risk of dental caries [4, 5]. Most of these risk factors for oral disease are behavioural and lifestyle related and are preventable through promotion of oral hygiene and oral health education [5].

Schools provide an opportunity for oral health promotion as children spend most of their time in school. Furthermore, schools provide links to the community, families, and dental and health care providers. School-based interventions can contribute to improving children’s oral health outcomes by addressing risk factors for oral diseases already at an early stage of life [6–9]. A range of school-based initiatives aimed at improving oral health includes integration of oral health within school health policies, provision of health-enabling environments and facilities, daily group tooth brushing, oral health education, parental and community involvement and active participation of children, screening, sealant, and fluoride varnish application programmes [1, 5, 10].

In 1995, the World Health Organisation (WHO) launched the Global School Health Initiatives aimed at spreading the health-promoting school approach worldwide [11].

One previous review of four randomised controlled trials assessed the clinical effects of primary school-based interventions aimed at changing behaviour in relation to tooth brushing habits and the frequency of consumption of cariogenic food and drink in 4–12-year-old children for prevention of caries [12]. The review was limited to changes in oral health outcomes from baseline and the conclusion was that there was insufficient evidence for the effectiveness of primary school-based behavioural interventions to reduce plaque and caries, and to improve oral health knowledge among 4–12-year-old children. Other reviews focused on the effect of health-educating and health-promoting interventions among children, teenagers, adults and seniors [13] and the effect of interventions based upon the health-promoting schools’ framework among children 4–18 years attending schools or colleges [14]. More information is needed on the effectiveness of primary school-based interventions in improving oral health.

Our systematic review aims to evaluate the effectiveness of school-based interventions in improving oral health compared to no intervention or usual practice among primary school children in LMICs—those countries with national income per person less than $12,375 [15]. We considered these countries because they experience a high burden of oral disease, and the risk of developing dental caries is high [3–5]. To the best of our knowledge, there is no previously published systematic review on this topic.

## Methods

The systematic review was conducted in accordance with the Joanna Briggs Institute (JBI) methodology for systematic reviews of effectiveness [16]. The title of this review was registered with PROSPERO (registration number: CRD42020202599).

### Eligibility criteria

This review considered studies that included children aged 3–16 years who attended primary school. We made post hoc changes to the age limits of participants included in this review because there were studies that had children in primary schools aged under 6 years and over 12 years.

We defined primary school-based interventions as comprising any one or more of the following elements: school health policy; provision of oral health education; promoting a healthy school environment; providing access to oral health services; and involving community members [17]. Studies were included if:

1. The intervention used schools as the focal site for intervention delivery and
2. Studies compared an intervention to no intervention or usual practice and
3. Studies were published in English from 1995 to December 2021 and
4. The intervention took place in a LMIC.

We included both experimental and observational studies. The primary outcome of interest was mean difference in dental caries between intervention and control group measured by decayed, missing, and filled teeth/surfaces [dmft(s)/DMFT(S)] scores. The dmft(s) is for primary dentition and DMFT(S) is for permanent dentition. The secondary outcomes included: difference in plaque, gingival disease, oral health knowledge, oral health attitude and oral health behaviour scores.

### Search strategy

A preliminary search of PROSPERO, MEDLINE, the Cochrane database of systematic reviews and the JBI database of systematic reviews and implementation reports was conducted and no ongoing systematic reviews on the topic were identified. Relevant databases were identified using the University of New South Wales (UNSW) Library’s subject guides. We identified all studies through an extensive search of MEDLINE, Embase, Global Health, CINAHL, Emcare, Scopus, Web of Science, the WHO website, Google Advanced and Google Scholar between 08/04/2020 and 07/06/2020. The following keywords and index terms were used with Boolean operators to combine searches: “oral health” OR "dental health" AND “school” AND “oral health promotion” OR "oral health education" AND “children “OR “child” AND “intervention” OR “effectiveness” AND “randomized controlled trial" OR "before and after study" OR “case–control study” OR “cohort study” OR “cross-sectional study”. The search strategy, including all identified keywords and index terms, were adapted for each included information source. The full electronic search strategy for MEDLINE is provided in Appendix 1. Additional search strategies included: (1) hand searching reference sections of included studies, (2) using the UNSW library to access articles unavailable online, and (3) we used automatic alerts of new results matching our strategy to update our search.

### Study selection

Following the search, all identified citations were collated and uploaded into Endnote and duplicates removed. Titles and abstracts were screened by one investigator (PA) for assessment against the inclusion criteria for the review. Potentially relevant studies were retrieved in full, and their citation details imported into COVIDENCE [18].

The full text of selected citations was assessed in detail against the inclusion criteria by PA and the other review team members (SK, RL, MJO, AES, RR) checked decisions for including studies. Any disagreements that arose between the reviewers was resolved through discussion.

### Data extraction

Two reviewers (AP and EO) independently used a standardised and piloted data extraction form. Data extraction from studies included: citation details, methodology, setting and context, population characteristics, intervention design, control design, and outcomes of significance for this review objective. We contacted authors of one study to request missing data.

### Quality assessment

Two reviewers (AP and EO) independently assessed for methodological quality using standardised instruments from the JBI for experimental and observational studies. The instrument for experimental studies had 13 domains, while the instrument for observational studies had 11 domains. Judgement was made by classifying domains as “yes”, “no”, “unclear” or “not applicable”. Any disagreements that arose were resolved through discussion. All studies, regardless of the results of their methodological quality, underwent data extraction.

### Data synthesis

Qualitative data are presented in narrative form, including tables to aid data presentation where appropriate. Quantitative data analyses were conducted in RevMan 5.4. for outcomes of significance for this review objective. Random-effects models were used for all meta-analyses. This approach allows for pooling of data accumulated from a series of studies with differences in subjects and interventions. Also, this approach allows for weighting of each trial, and provides a mean difference score between intervention and controls and confidence interval (CI) that represent all the trials included in a given analysis. Standardized mean difference (SMD) scores (rather than raw mean scores) were used in meta-analyses to account for heterogeneity among extracted measures. We used risk ratios (RR) in one meta-analysis.

### Sensitivity analysis

We planned a sensitivity analysis to test decisions made regarding computation of meta-analysis with and without the inclusion of poor-quality studies, inclusion of studies with small sample size and the use of both random effects and fixed effect models. The review team planned a subgroup analysis to address whether the effect measures vary in relation to specific characteristics of the included studies or their participants such as age of participants, region of study, different elements in the intervention, and frequency and duration of exposure to intervention. Planned assessment of publication bias using funnel plots was found inappropriate because there were less than 10 studies included in each meta-analysis.

### Certainty of evidence

The Grading of Recommendations, Assessment, Development and Evaluation (GRADE) approach for grading the certainty of evidence was followed [19] and a Summary of Findings (SoF) created using GRADEPro GDT (XX/2014) [20].

## Results

**Fig. 1:**
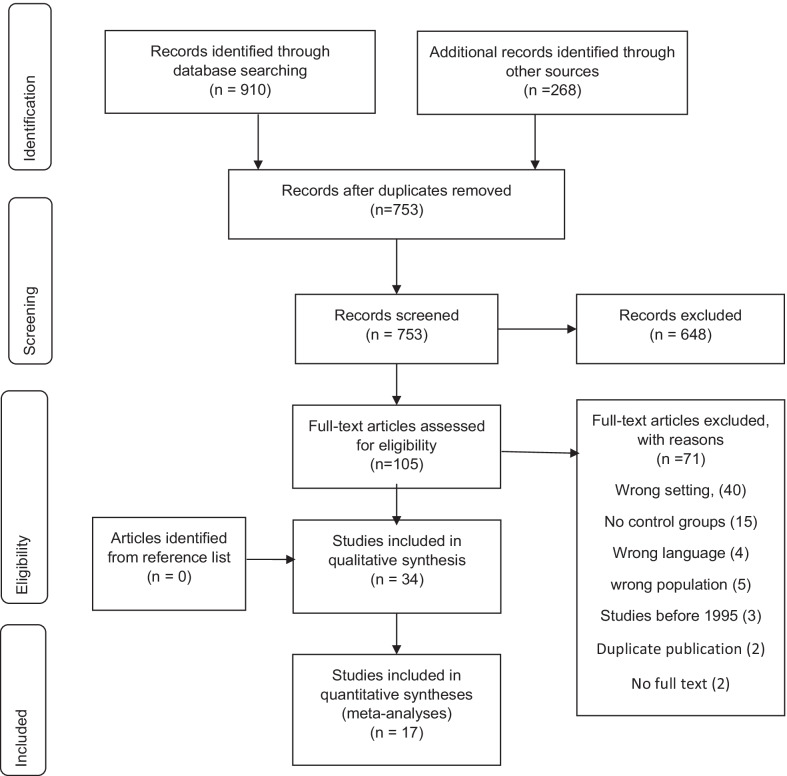
Results of the search presented in a Preferred Reporting Items for Systematic Reviews and Meta-analyses (PRISMA) flow diagram

Results of the search are presented in a preferred reporting items for systematic reviews and meta-analyses (PRISMA) flow diagram (Fig. 1) and a PRISMA checklist is provided as an additional file [21] (Additional file 1).

The search yielded 1178 publications, 910 through database searching and 268 were identified from other sources. After removing duplicates 753 remained. A further 648 were excluded after screening titles and abstracts. This review included 34 articles after excluding 71 full text articles. Details of excluded studies and reasons for exclusion are reported in Additional file 2.

### Study characteristics

This review included 30 experimental studies and 4 observational studies that were conducted in LMICs and published between 1996 and December 2021. Eighteen studies were conducted in Southeast Asia, 6 in Africa, 5 in the Americas, 2 in Europe, and 3 in eastern Mediterranean.

Most interventions were oral health education programs with various activities, however, five interventions exclusively involved disclosed plaque visualisation [22], daily tooth brushing at school [23, 24], application of fissure sealants [25], and zinc supplementation [26]. Three oral health education programs were based on the WHO health promoting school concept [8, 9, 27]. Other studies incorporated one or more of the activities of interventions listed in Table 1.

**Table 1:** List of activities included in interventions/studies

No	List of activities included in interventions/studies
01	Oral health education sessions for children [8, 28–49]
02	Oral health education sessions for parents [27, 28, 30, 32, 33]
03	Integration of activities into the school curriculum [8, 28, 31, 33, 34, 50, 36]
04	Training for those delivering the program [8, 27, 28, 31–33, 50, 51, 45–47, 50–52] and follow up training for reinforcement [8, 27]
05	Provision and use of educational materials to children such as charts, posters, pictures, games, models and audio-visual aids [8, 9, 27, 29, 30, 32–34, 51, 40–45, 44, 45]
06	Demonstration of oral health self-care [27, 31, 34, 38, 40, 44–46]
07	Provision and use of instruction manuals [8, 31, 33, 46–48, 48]
08	Oral examination, informing parents [9, 25, 32, 34, 37, 43] and offer of free treatment [37]
09	Provision of fluoride toothpaste [9, 33, 34, 52] and tooth brush [28, 34]
10	Provision of fluoride mouth rinse [38, 45] and fluoride gel [38]
11	Regular visits to motivate teachers [27, 34]
12	Supervised tooth brushing [23, 24, 27, 31, 50, 52, 38, 39] and flossing [39], and brush with disclosed plaque visualization [22]
13	Tour of dental hospital/health clinic [9, 36]
14	Peer health education, supervision of toothbrushing and training of children [36]
15	Provision of sugar free chewing gum [27]
16	Knowledge contests on oral health knowledge, painting oral health situations, brushing teeth, and public speaking [9, 29, 30, 36]
17	Provision of preventive and curative care including sealing pits and fissures, removal of calculus, restoring cavities, emergency care, treating pulpally involved teeth, and zinc supplementation [9, 25, 26, 50, 52]
18	Provision of take-home materials such as brush charts, parent educational flyers and calendars [30, 33, 43, 53, 48]
19	Use of mass media to provide information about the project activities [31, 50, 52]
20	Attending annual conferences [39] and national convention and exhibition [36]
21	Action planning exercises, rate how confident they were and identify barriers to dental flossing [35]
22	Children conduct health activities at school [36, 42, 47]
23	Children formulate a proposition of, ways to overcome obstacles and formulate goals to brush three times a day [44]
24	Demonstration and practice on how to make a chew stick [46]

Interventions had varied durations and ranged from a few minutes of brushing with disclosed plaque visualisation [22], to a seven-year fissure sealant program [25]. Nineteen interventions were conducted between one month and one year [24, 26, 29, 30, 32–35, 37, 40–47, 50, 53] while four interventions were performed for over three years [25, 28, 36, 51].

Most interventions were delivered by either a dentist, teacher, or dentist and teacher combination, while others were delivered by investigators, health counsellors, community members, parents, and school children. Three studies did not include information on the persons that delivered the interventions [35, 38, 40].

Twenty-three studies included in this review had two intervention arms, seven studies had three arms [24, 27, 38, 41, 44, 46, 48], three had four arms [30, 43, 45], and one had five arms [49]. Comparators in all studies were schools that did not receive an intervention or continued to provide usual activities.

The most frequently reported outcome measures were periodontal disease and dental caries. The least reported outcomes were consumption of sugar sweetened foods and beverages, oral health care utilization, quality of life and odontogenic infections. None of the studies reported any adverse outcomes.

Twenty-four studies were classified as cluster randomised controlled trials [8, 9, 22, 24, 25, 27–31, 34, 37, 41–51, 53], two non-randomised trials [23, 32], four quasi-experiments [25, 33, 40, 52] and four cohort studies [24, 36, 38, 39]. Additional file 3 shows characteristics of the included studies in more detail.

### Quality assessment

The studies included in our systematic review varied in quality of methodology. None of the experimental studies scored a “yes” for all 13 domains assessed. Experimental studies showed limitations with respect to randomisation of participants, allocation concealment, blinding of participants, persons delivering the intervention and outcome assessors, intention to treat analysis, statistical power analysis and trial design. Details of the assessment are presented in Table 2.

**Table 2:** Results of assessment of methodological quality of eligible experimental and quasi-experimental studies

S/N	Study	Quality assessment domains															
		1	2	3	4	5	6	7	8	9	10	11	12a	12b	12c	12d	13
1	Chachra et al. [45]	3	3	2	3	2	3	1	3	3	1	3	3	3	2	1	2
2	Chauhan et al. [30]	3	3	1	1	2	1	1	2	2	1	1	1	1	1	1	2
3	Chounchaisithi et al. [22]	3	1	1	2	2	1	1	2	2	1	1	1	3	1	1	2
4	De Farias et al. [29]	3	3	1	3	2	3	1	1	2	1	1	1	3	1	1	2
5	Duijster et al. [23]	2	3	1	3	2	1	1	1	2	1	1	1	1	1	1	1
6	Esan et al. [28]	1	1	3	3	2	3	2	3	3	1	3	1	3	1	1	2
7	Frencken et al. [51]	3	3	1	3	2	2	2	1	2	1	1	1	3	1	1	2
8	Gholami et al. [35]	3	3	1	3	3	3	1	1	1	1	3	1	3	1	1	2
9	Haleem et al. [49]	1	1	2	1	2	1	1	1	2	1	1	1	1	1	1	1
10	Hartono et al. [31]	3	3	2	3	2	2	1	1	1	1	3	1	3	1	1	2
11	Hebbal et al. [41]	1	3	1	3	2	1	1	3	3	1	1	1	3	1	1	2
12	Hebbal and Nagarajappa [37]	1	3	1	3	2	3	1	3	2	1	3	1	3	2	1	2
13	Jaime et al. [32]	2	2	2	3	2	3	1	1	2	1	3	1	3	1	1	2
14	Kapadia et al. [33]	2	3	1	3	3	1	1	3	3	1	3	1	3	1	1	2
15	Naidu and Nandlal [34]	3	3	1	3	2	2	1	2	2	1	1	1	3	1	1	2
16	Nammontri et al. [42]	1	1	1	3	2	3	1	1	2	1	1	1	3	1	1	1
17	Nyandindi et al. [46]	3	3	3	3	2	1	1	1	1	1	1	1	3	1	1	2
18	Pakhomov et al. [52]	2	2	1	3	2	1	1	2	2	1	1	1	3	1	1	2
19	Peng et al. [27]	3	3	2	3	2	3	1	1	2	1	1	1	3	1	1	2
20	Petersen et al. [8]	3	3	1	3	2	3	1	1	2	1	1	1	3	1	1	2
21	Saied-Moallemi et al. [43]	3	3	2	1	2	1	1	1	2	1	1	1	1	1	1	1
22	Simpriano and Mialhe [44]	3	3	1	3	2	3	1	1	2	1	3	1	1	1	1	2
23	Swe et al. [40]	3	3	2	3	3	3	1	1	1	1	3	1	3	1	1	2
24	Tai et al. [9]	1	1	1	3	2	1	1	1	2	1	1	1	1	1	1	1
25	Tomazoni et al. [47]	1	1	2	3	2	3	1	1	2	1	1	1	1	1	1	1
26	Uckardes et al. [26]	3	3	1	1	1	1	1	1	2	1	3	1	3	3	1	2
27	Van Palenstein et al. [50]	3	3	1	3	2	1	1	1	2	1	1	1	3	1	1	2
28	Van Wyk et al. [25]	1	3	1	3	2	3	1	2	2	1	3	1	3	1	1	1
29	Yekaninejad et al. [48]	3	3	1	3	2	3	1	1	1	1	1	1	1	1	1	1
30	Zacharias et al. [53]	3	1	2	1	2	2	1	1	2	1	3	1	1	1	1	1

Key: 1 Yes; 2 No; 3 Unclear

Domain 1: Was true randomization used for assignment of participants to treatment groups?

Domain 2: Was allocation to treatment groups concealed?

Domain 3: Were treatment groups similar at the baseline?

Domain 4: Were participants blind to treatment assignment?

Domain 5: Were those delivering treatment blind to treatment assignment?

Domain 6: Were outcomes assessors blind to treatment assignment?

Domain 7: Were treatments groups treated identically other than the intervention of interest?

Domain 8: Was follow up complete and if not, were differences between groups in terms of their follow up adequately described and analysed?

Domain 9: Were participants analysed in the groups to which they were randomized?

Domain 10: Were outcomes measured in the same way for treatment groups?

Domain 11: Were outcomes measured in a reliable way?

Domain 12a: Was appropriate statistical analysis used? Checked if the assumptions of statistical tests were respected

Domain 12b: Was appropriate statistical analysis used? Checked if appropriate statistical power analysis was performed

Domain 12c: Was appropriate statistical analysis used? Checked if appropriate effect sizes were used;

Domain 12 d: Was appropriate statistical analysis used? Checked if appropriate statistical procedures or methods were used given the number and type of dependent and independent variables, the number of study groups, the nature of the relationship between the groups (independent or dependent groups), and the objectives of the statistical analysis (association between variables; prediction; survival analysis)

Domain 13: Was the trial design appropriate for the topic, and any deviations from the standard RCT design (individual randomization, parallel groups) accounted for in the conduct and analysis of the trial?

Observational studies had limitations regarding strategies to deal with confounding factors, participants without outcome of interest at start of the study, and strategies to address incomplete follow up. Details of the assessment are presented in Table 3.

**Table 3:** Results of assessment of methodological quality of observational studies using standardised critical appraisal instruments from the Joanna Briggs institute

Study	Quality assessment domains										
	1	2	3	4	5	6	7	8	9	10	11
de Sousa et al. [52, 38]	1	1	1	1	1	3	1	1	1	2	1
Lai et al. [37, 39]	1	1	1	1	1	3	1	1	1	2	1
Monse et al. [22, 24]	1	1	1	1	2	2	1	2	1	2	2
Yusof et al. [50, 36]	1	1	1	1	1	3	1	1	1	2	1

Key: 1 Yes; 2 No; 3 Unclear

Domain 1: Were the two groups similar and recruited from the same population?

Domain 2: Were the exposures measured similarly to assign people to both exposed and unexposed groups?

Domain 3: Was the exposure measured in a valid and reliable way?

Domain 4: Were confounding factors identified?

Domain 5: Were strategies to deal with confounding factors stated?

Domain 6: Were the participants free of the outcome at the start of the study?

Domain 7: Were the outcomes measured in a valid and reliable way?

Domain 8: Was the follow up time reported and long enough for outcomes to occur?

Domain 9: Was follow up complete, and if not, were the reasons to loss to follow up described and explored?

Domain 10: Were strategies to address incomplete follow up utilized?

Domain 11: Was appropriate statistical analysis used?

### Effectiveness of interventions compared with controls

The reported primary school-based interventions were effective in decreasing dental caries [8, 9, 23, 25, 27, 34, 38, 39, 45, 52], improving gingival health [8, 9, 27, 29, 30, 34, 42, 43, 48, 49, 53], reducing plaque [9, 22, 29, 31, 33, 34, 39, 41, 43, 44, 46, 49], improving oral health knowledge [8, 29, 31, 32, 34, 40, 44, 46, 49], improving attitudes to oral health [34, 46], improving oral health practices [8, 9, 32, 34, 35, 37–40, 46, 48, 49, 53], and improving oral health related quality of life [36, 42, 47].

Details of effectiveness of interventions compared with controls are presented in Additional File 4.

#### Oral health education programs

One component common in 27 reports of effective interventions was an oral health education program [8, 9, 27–49, 52, 53]. Studies that incorporated skills-based education using educational methods such as lessons, demonstrations, supervised tooth brushing, peer teaching, field trips and active participation using educational materials such as booklets, posters, audio-visual aids and models, reported positive effects on plaque outcomes [9, 29, 31, 33, 34, 39, 41, 43, 46, 49], gingival health outcomes [29, 42, 43, 48], oral health behaviour [9, 28, 31, 32, 35, 36, 40, 48, 49], dental caries [9, 34, 39, 45], oral health knowledge [31, 32, 40, 41, 44, 46], oral health attitude [46], oral health related quality of life [42, 47], and oral health belief [42]. Skills based education in a few studies involved integration of activities into the school curriculum [8, 28, 31, 33, 34, 36, 50].

On the other hand, studies that involved oral health education programs showed no significant difference between the intervention and control groups for dental caries outcomes [31, 32, 50, 51] and plaque outcomes [30, 50, 51, 53], gingival health outcomes [50] and oral health attitude outcomes [32].

Among studies that had more than one intervention arm: non-significant improvement in plaque outcomes were reported for oral health education by teachers, or peers and a self-learning group [49], oral health education without audio visual aids [41], and oral health education via class work, parents and both class work and parents [30]; non-significant improvement in gingival health outcomes were reported for oral health education via both class work, parents and children [30], self-learning group [49]; non-significant improvement in oral health knowledge and practice outcomes were reported for oral health education in a self-learning group [49] and conventional group [46].

#### Training of those delivering interventions

Studies that incorporated training of those delivering interventions on topics such as the importance of oral health, causes and prevention of oral disease, oral anatomy and tooth development, diet and nutrition, importance of dental visits and emergency oral care at school and training on methods of providing oral health education were effective in reducing dental caries [8, 27, 34, 52], reducing plaque [31, 49], improving gingival health [8, 27, 42], adopting healthy practices such as regular tooth brushing and use of fluoridated toothpaste [8, 32, 34, 36, 46], changing attitude regarding sweets as harmful to teeth and having positive attitude towards the treatment for dental decay [34, 46], and improving oral health related quality of life, sense of coherence and oral health beliefs [42]. However, some studies that incorporated training of those delivering the intervention showed no significant difference between the intervention and control groups for DMFS/DMFT increment [8], mean dmft/dmfs [31] and DMFT [32] scores, oral health attitudes and practices [32], mean oral impacts on daily performance (OIDP) score and prevalence of OIDP [36].

#### Provision of oral health services

A few studies incorporated the provision of access to school health services, including oral health examination and providing a report on the dental status of children to parents, a fissure sealant program, provision of treatment including sealing pits and fissures, removal of calculus, restoring cavities and treating pulpally involved teeth, and providing fluoride mouth gel and rinse. These studies showed reduced dental caries and plaques scores, and improved oral health practices, gingival health and knowledge [9, 34, 38, 45].

#### Engaging parents

Eleven studies that engaged parents by providing oral health education sessions, delivering the interventions to children or providing reports on oral health status showed positive effects for oral health outcomes [8, 9, 27, 28, 30, 32, 33, 43, 46, 48, 53]. The studies showed positive effect for mean increment of fs/FS [8, 27], mean increment in DMFS scores [9, 27], gingival health [8, 9, 27, 30, 32, 43, 48, 53], plaque scores [9, 33, 43], oral health attitudes such as regarding sugar-containing foods as harmful [46], oral health practices such as dental visits [8, 9, 28] use of fluoridated toothpaste [8, 9, 28], consuming sugar-containing foods and drinks [8, 28, 32, 46] receiving restorations and sealants [9], flossing [28, 32, 48], tooth brushing [9, 28, 46, 48, 53], and skills in making chew sticks [46]. However, 3 studies that engaged parents in interventions showed no significant difference between the intervention and control groups for mean DMFT scores [32], plaque scores [30, 32, 53] and oral health attitude [32].

#### Changes in school environment

Eleven studies that incorporated changes to the school environment through means such as hanging posters, tour of hospitals, providing free toothbrushes and toothpaste, working on healthy school projects, action planning exercises and attending conferences, reported a positive effect on oral health outcomes such as, mean increment in fs/FS scores [8, 27], mean increment in DMFT/S scores [9, 27, 52], dental caries in primary teeth [34, 52], gingival health [8, 9, 27, 32, 34, 42, 49], plaque scores [9, 33, 49] and oral health practices like dental visits [8, 9] use of fluoridated toothpaste [8, 9], consumption of sugar-containing foods and drinks [8, 32, 34], receiving restorations and sealants [9], tooth brushing [9, 34] and flossing [32, 35], oral health behaviour and knowledge scores [49], positive attitude towards the treatment for tooth decay [34], oral health beliefs [42] and oral health related quality of life [42, 47]. However, 1 study that incorporated changes in school environment in interventions showed no significant difference between the intervention and control groups for mean DMF, plaque and oral health attitude scores [32].

#### Based on WHO health promoting schools concept

All studies based on the WHO health promoting schools concept were effective in reducing dental caries [8, 9, 27], improving gingival health [8, 9, 27] and improving oral health practices like dental visits [8, 9] use of fluoridated toothpaste [8, 9], tooth brushing [9], consumption of sugar-containing foods and drinks [8], and receiving restorations and sealants [9].

#### Other interventions

Other effective interventions were plaque visualisation [22], daily tooth brushing as a group activity [23], and a fissure sealant program [25]. One study that included zinc supplementation alone [26], reported no significant difference between the intervention and control groups for plaque outcomes and gingival health outcomes.

### Meta-analysis

We performed meta-analyses to determine the effect of interventions on changes in DMFT/S, net DMFT/S increment, plaque, and gingival scores based on different intervention strategies. Selected studies varied in their primary outcomes, therefore, data from studies with similar outcomes were pooled and analysed.

### Dental caries measured by DMFT scores

Data from four cluster randomised trials, two quasi experiments and one non-randomized clustered controlled trial (n = 6766 participants) were pooled to determine the effects of school-based intervention strategies on the changes of DMFT scores.

**Fig. 2:**
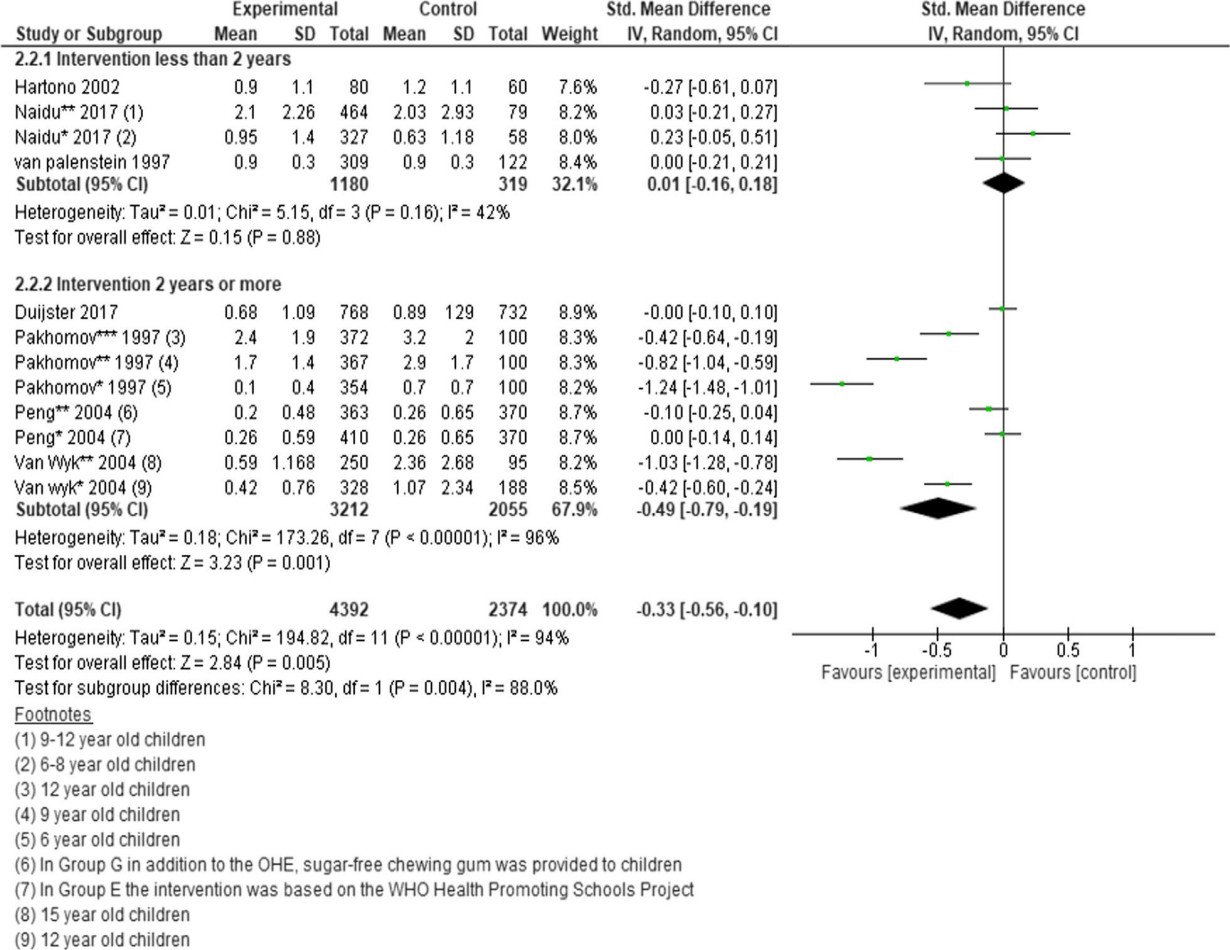
Forest plot of the effect of school-based intervention on dental caries by measurement of DMFT scores

However, one of the studies was a 3-arm RCT and three studies presented data for different age groups, therefore each intervention and age group were analysed separately. A statistically significant difference was found favouring interventions vs. controls (SMD =  − 0.33; 95% CI − 0.56 to − 0.10; *P* = 0.005) (Fig. 2) [23, 25, 27, 31, 34, 50, 52].

### Dental caries measured by net increment in DMFT scores

**Fig. 3:**
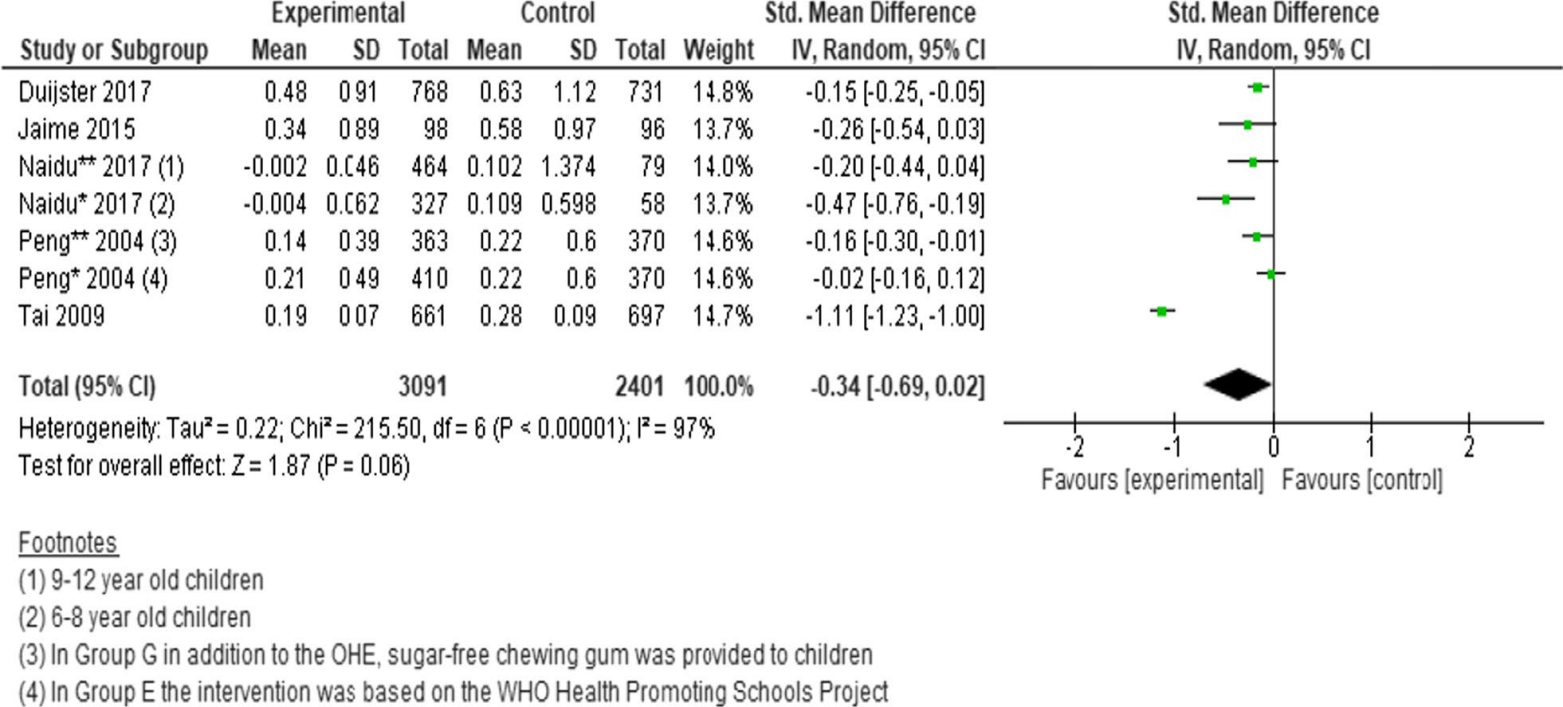
Forest plot of the effect of school-based intervention on dental caries by measurement of net increment in DMFT scores

Three cluster randomised trials and two non-randomized trials (n = 5492 participants) were pooled to determine the effects of interventions on the changes in net increment in DMFT scores. Since one of the studies was a 3-arm RCT and one study presented data for different age groups, we analysed each intervention and age group separately. Despite an overall tendency to favour the intervention group, no statistically significant difference was found between intervention and controls (SMD =  − 0.34; 95% CI − 0.69 to 0.02; *P* = 0.06) (Fig. 3) [9, 23, 27, 32, 34].

### Dental caries measured by DMFS scores

**Fig. 4:**
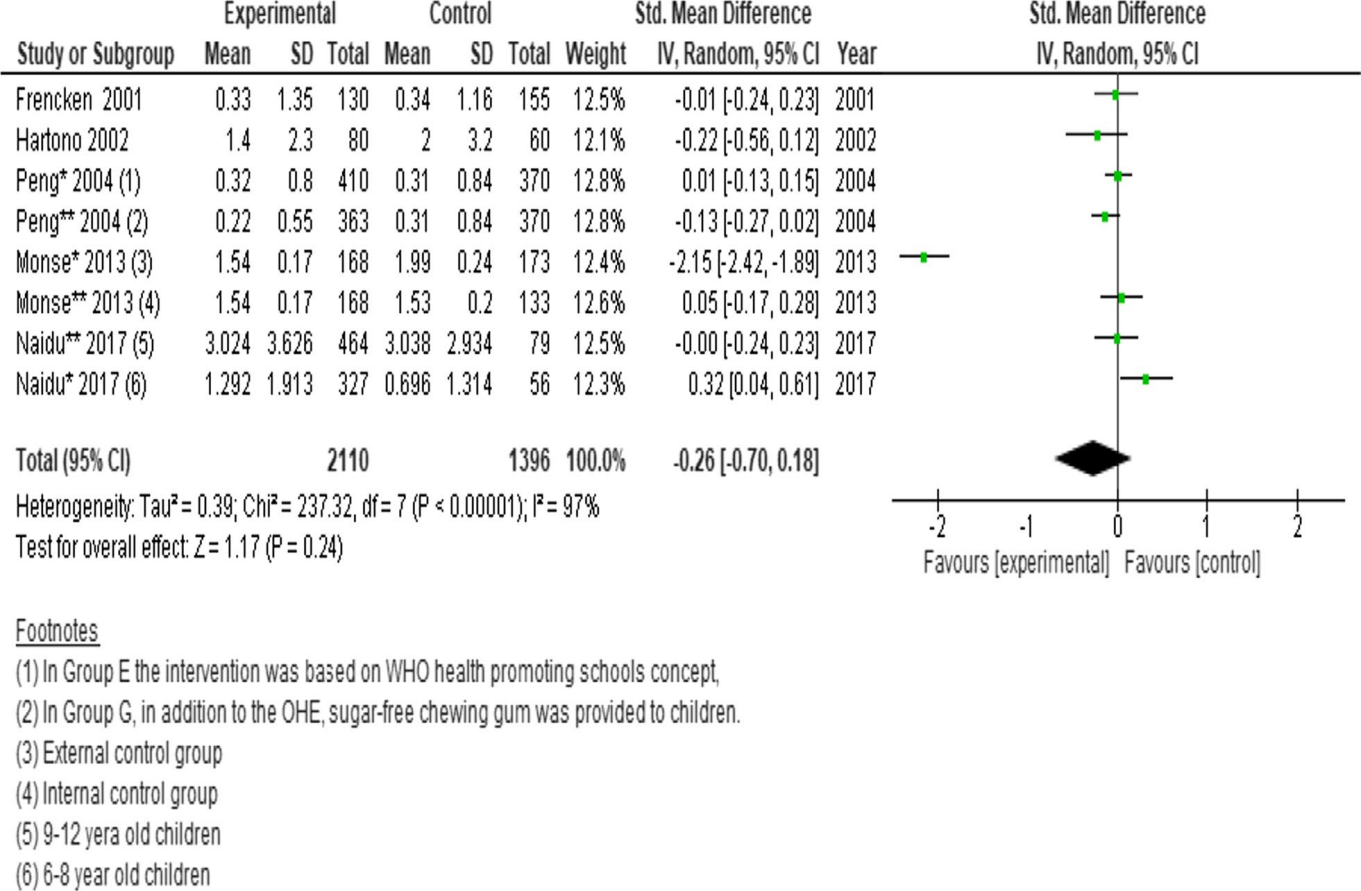
Forest plot of the effect of school-based intervention on dental caries by measurement of DMFS scores

Pooled data from four cluster randomised trials and a longitudinal cohort (n = 3506 participants) showed no significant difference in the changes of DMFS scores between intervention and control. (SMD =  − 0.26; 95% CI − 0.70 to 0.18; *P* = 0.24) (Fig. 4) [24, 27, 31, 34, 51].

### Dental caries measured by net increment DMFS scores

**Fig. 5:**
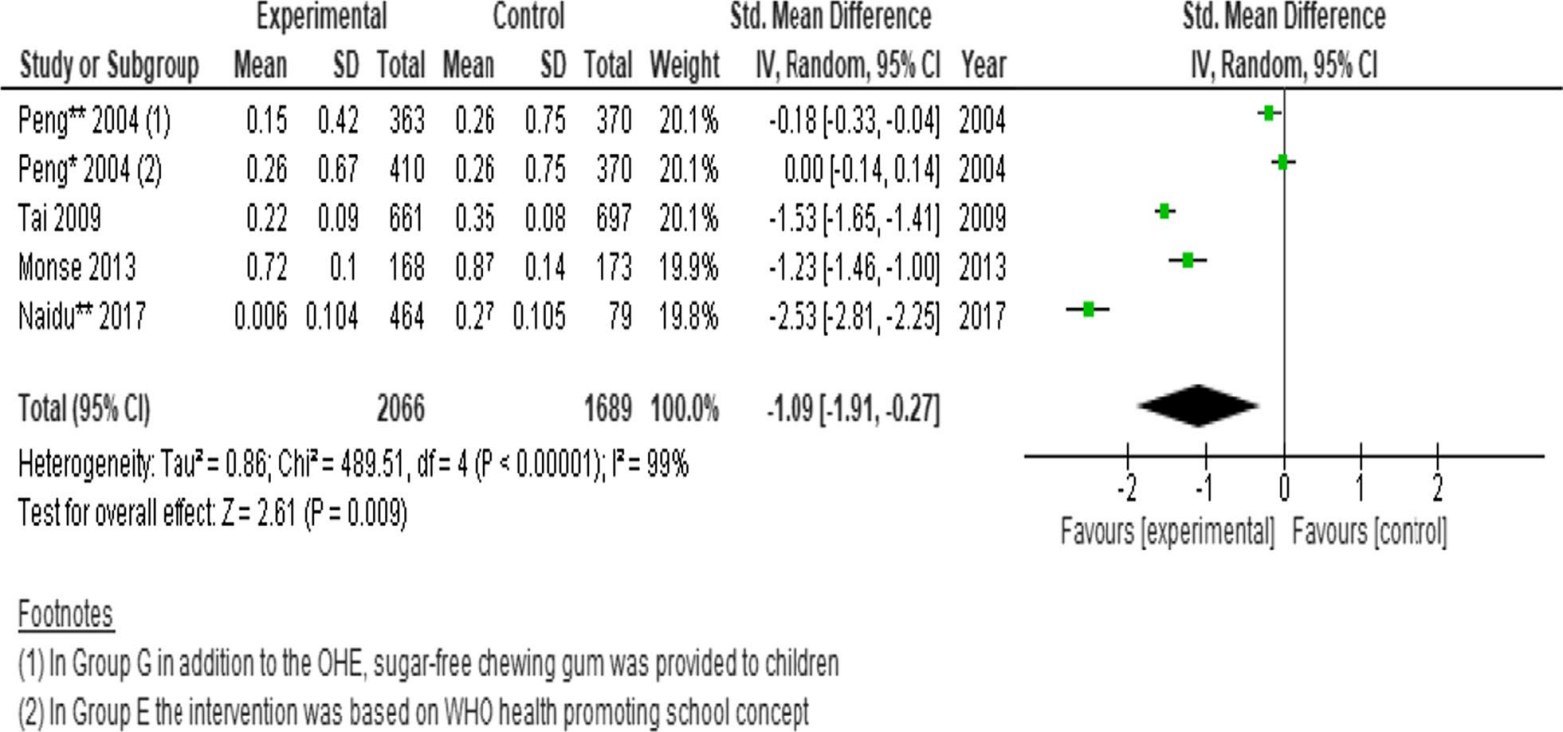
Forest plot of the effect of school-based intervention on dental caries by measurement of net increment in DMFS scores

Three cluster randomised trials and a longitudinal cohort (n = 3755 participants) were pooled to determine the effects of interventions on the changes in net increment in DMFS scores. As one of the studies was a 3-arm RCT, we analysed each intervention separately. A statistically significant difference was found favouring the intervention. (SMD =  − 1.09; 95% CI − 1.91 to − 0.27; *P* = 0.009) (Fig. 5) [9, 24, 27, 34].

### *Dental caries prevalence measured by dmft and DMFT/S score* > *1*

**Fig. 6:**
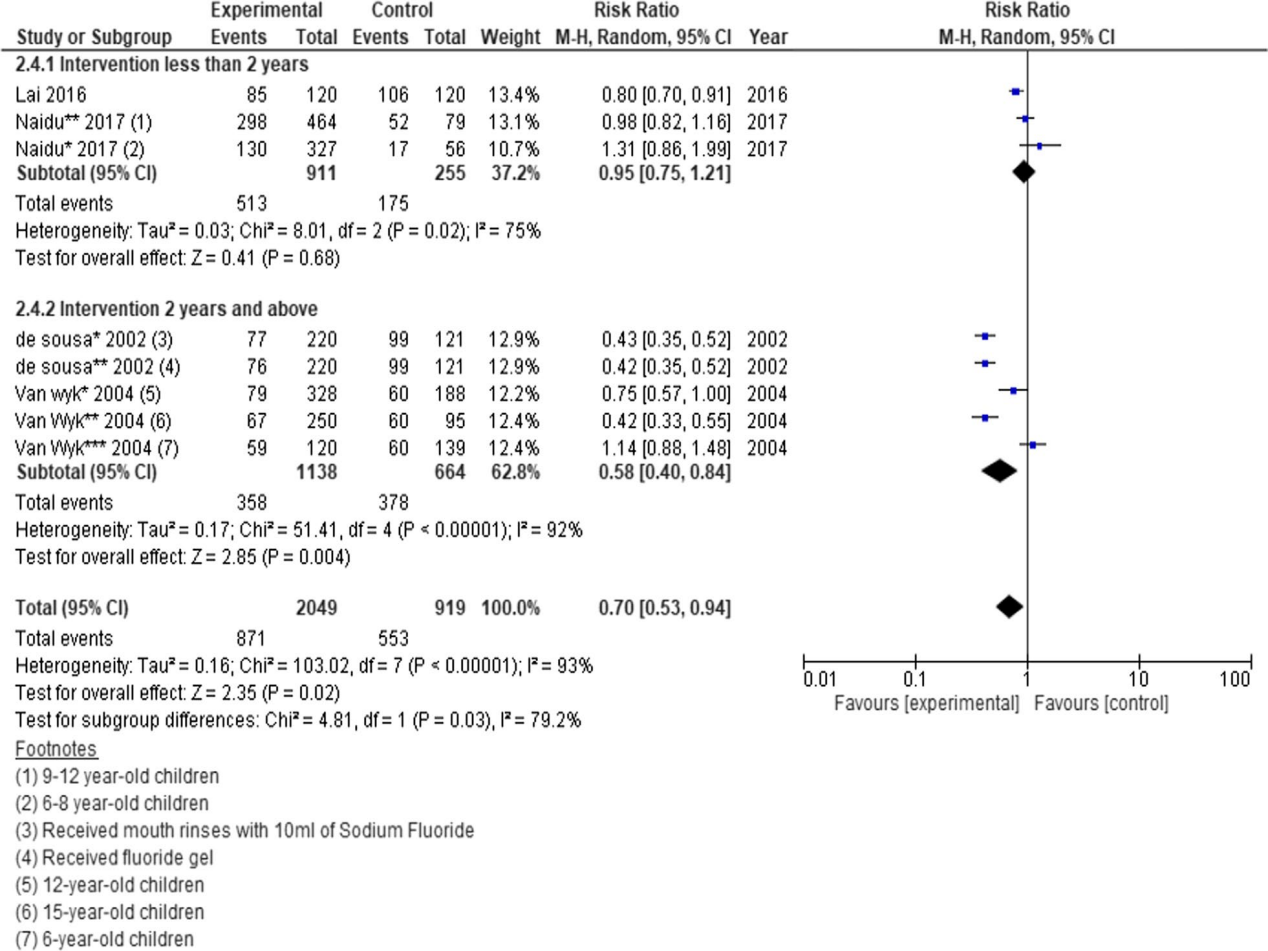
Forest plot of the effect of school-based intervention on dental caries prevalence by measurement of dmft or DMFT/S score greater than one

Two cluster randomised trials, a prospective cohort, and a retrospective cohort (n = 2968 participants) were pooled to determine the effects of school-based interventions on the changes in prevalence of dental caries. However, one of the studies was a 3-arm cohort and two studies presented data for different age groups. We thus analysed each intervention and age group separately. A statistically significant risk ratio was found favouring the intervention. (RR = 0.70; 95% CI 0.53 to 0.94; *P* = 0.02) (Fig. 6) [25, 34, 38, 39]. Fewer than five studies presented data on dental caries experience measured by dmft and dmfs indices, therefore those effects were not estimated.

### Oral hygiene measured by plaque scores

**Fig. 7:**
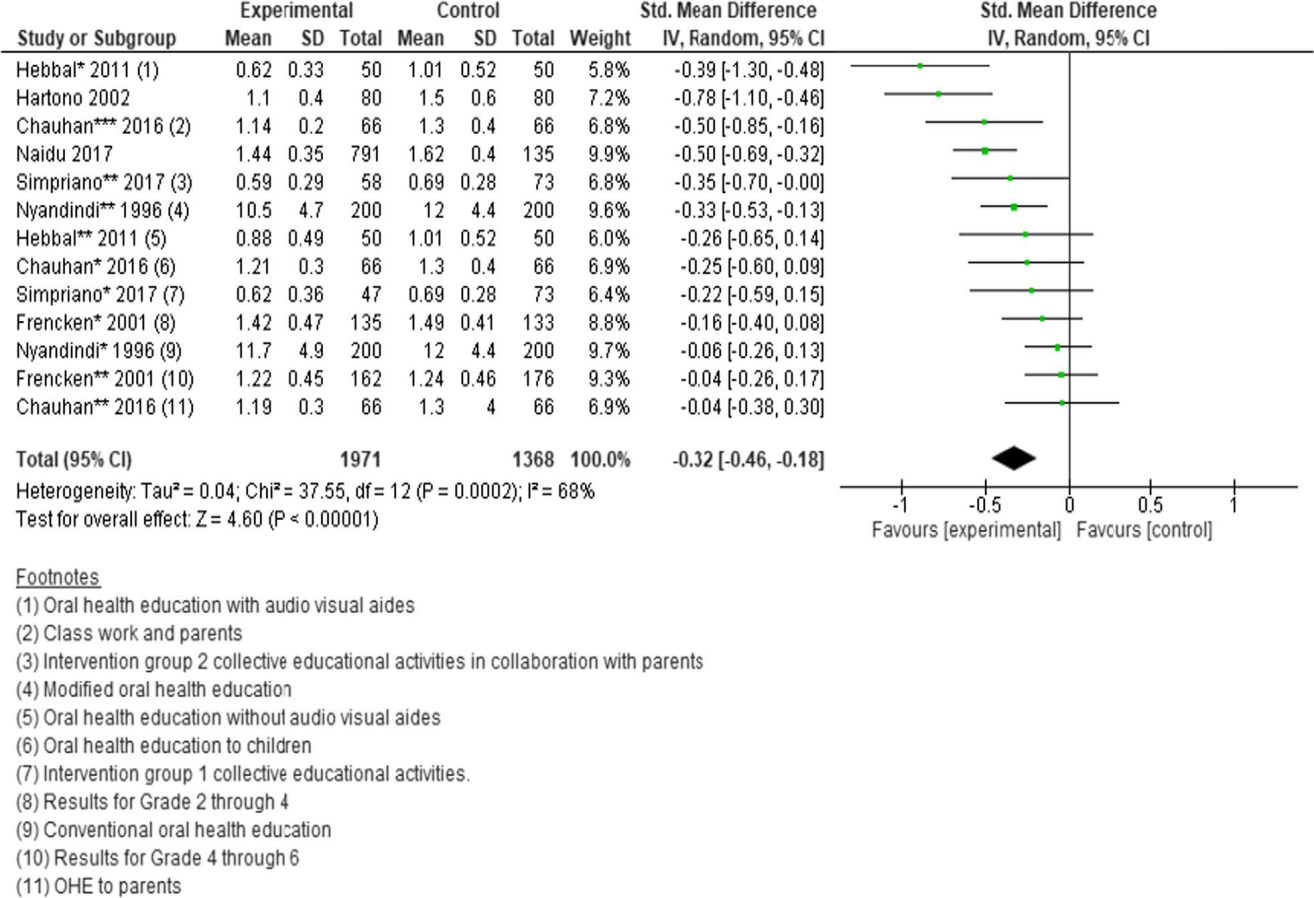
Forest plot of the effect of school-based intervention on oral hygiene by measurement of plaque scores

Six cluster randomised trials and a quasi-experimental study (n = 3339 participants) were pooled to determine the effects of interventions on the changes in plaque scores. However, four of the studies were a 3-arm trials and one presented data for different classes. We thus analysed each intervention and class separately. After pooling data, the standard mean difference in plaque scores favoured interventions compared to controls. (SMD =  − 0.32; 95% CI − 0.46 to − 0.18; *P* < 0.00001) (Fig. 7) [30, 31, 34, 41, 44, 46, 51]. Fewer than three studies presented data on proportions of those with or without plaque, plaque score reductions, plaque score increments, percentage score and plaque score with 95% CI, therefore those effects were not estimated.

### Oral hygiene measured by mean gingival scores

**Fig. 8:**
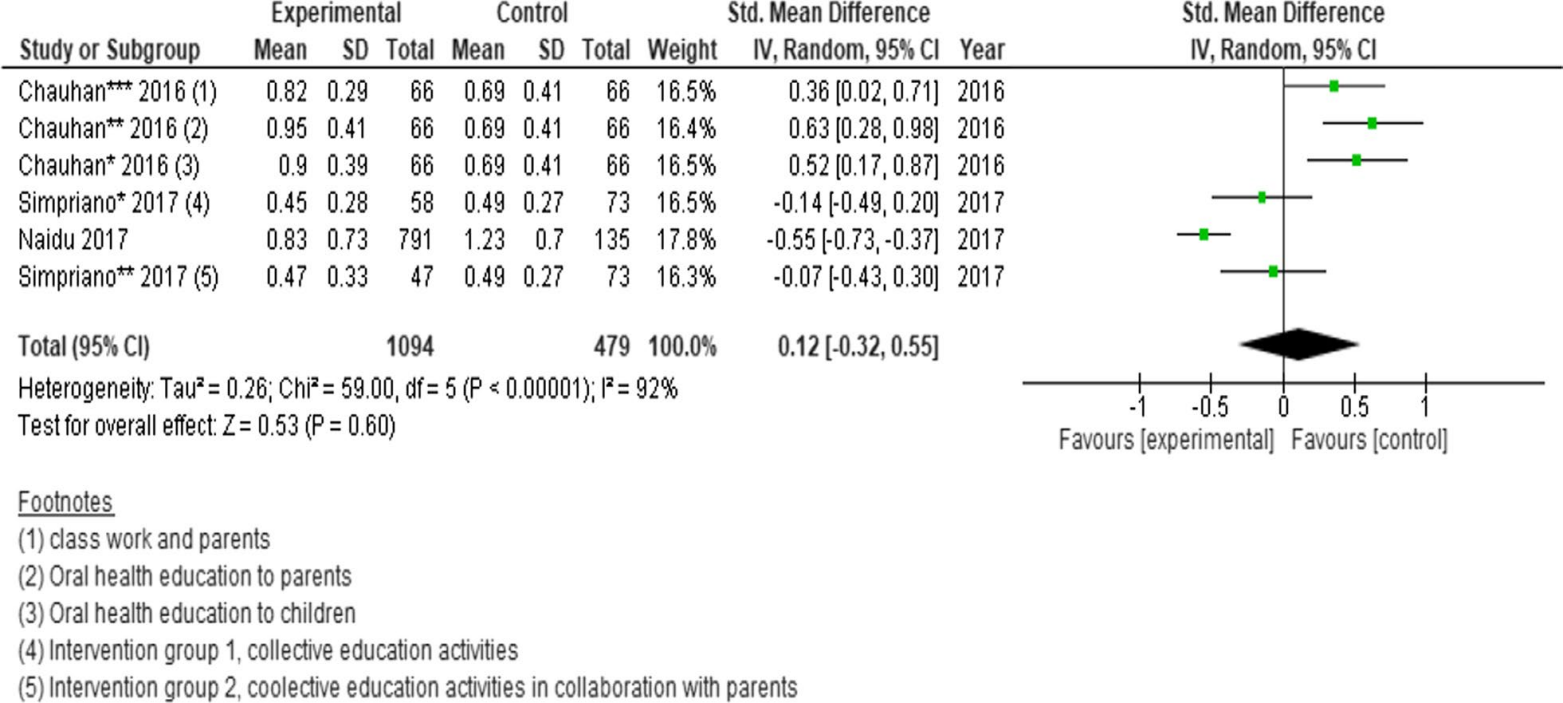
Forest plot of the effect of school-based intervention on oral hygiene by measurement of mean gingival scores

Three randomised controlled trials (n = 1573 participants) were pooled to determine the effects of interventions on the changes in gingival scores. However, two of the studies had multiple interventions and each arm intervention was analysed separately. No statistically significant difference was found between intervention and controls (SMD = 0.12; 95% CI − 0.32 to 0.55; *P* = 0.60) (Fig. 8) [30, 34, 44]. Fewer than three studies presented data bleeding increments, proportion of persons with decreased or increased gingival scores, and proportion of persons satisfactory or unsatisfactory, therefore those effects were not estimated.

### Sensitivity analysis

We conducted sensitivity analysis by dropping studies of poor quality and small sample size; use of both random effects and fixed effect models and relative risk and odds ratios to examine the influence on the summary effect estimates. For all meta-analyses results differed across sensitivity analyses (Additional file 5).

### Subgroup analysis

For each meta-analysis the studies were divided into various subgroups as we found appropriate. The subgroups included intervention type, intervention duration and age of participants. We found standardised mean difference in DMFT scores was significantly higher in interventions that lasted more than two years compared to interventions less than two years, − 0.49 (95% CI − 0.79 to − 0.19; *P* = 0.001) versus 0.01 (95% CI − 0.16 to 0.18; *P* = 0.88); *P* = 0.004 (Fig. 2). Relative risk of dental caries was also significantly lower among children who received interventions that lasted two years or more compared to interventions that lasted less than two years 0.58 (95% CI 0.40–0.84; *P* = 0.004) versus 0.95 (95% CI 0.75–1.2; *P* = 0.68) (Fig. 6).

We found no significant subgroup difference in DMFT/S scores, net increment in DMFT/S scores, dmft or DMFT/S score > 1 between oral health education interventions and other interventions (daily tooth brushing, fissure sealant). For example, SMD of net increment in DMFS scores for oral health education interventions [9, 27, 34] was − 1.05 (95% CI − 2.03, − 0.08; *P* = 0.03) compared to -1.23 (95% CI − 1.46, − 1.00; *P* < 0.00001) for other interventions (daily tooth brushing) [24]. Test for subgroup differences: Chi^2^ = 0.11, df = 1 (*P* = 0.74), I^2^ = 0%.

No significant subgroup difference in DMFT and net increment in DMFT/S scores between interventions based on WHO framework and other interventions. For example, the SMD of net increment in DMFS scores for interventions based on the WHO framework [9, 27] was − 0.57 (95% CI − 1.56, 0.42; *P* = 0.26) compared to -1.88 (95% CI − 3.15, − 0.60; *P* = 0.004). Test for subgroup differences: Chi^2^ = 2.50, df = 1 (*P* = 0.11), I^2^ = 60.0%. Also. SMD in DMFs scores was not different between in interventions with three or less activities compared to interventions with more than three activities less than two years, 0.58 (95% CI − 1.62, 0.45; *P* = 0.27) versus 0.02 (95% CI − 0.13, 0.17; *P* = 0.80); *P* = 0.26, I^2^ = 21.2%.

We found that the standardised mean difference in DMFT scores was significantly higher in interventions among children 8–15-years-old compared to interventions among children 6–8-years-old, − 0.41(95% CI − 0.50, − 0.32; *P* < 0.00001) versus − 0.11(95% CI − 0.17, − 0.04; *P* = 0.001); *P* < 0.00001, I^2^ = 96.6%. On the other hand, there was no significant subgroup difference standardised mean difference in net increment DMFT and DMFS scores and RR between interventions involving children 5–8 years versus 9–12 years, 6–8 years versus 9–12 years and 6–8 years versus 9–15 years, respectively. For example, the SMD of net increment in DMFT scores for interventions invoking children 5–8 years old [9, 23, 27, 32, 34] was − 0.36 (95% CI − 0.76, 0.04; *P* = 0.07) compared to − 0.20 (95% CI − 0.44, 0.04; *P* = 0.10) for 9–12 year-old children [34].

### Certainty of evidence

Certainty of evidence was assessed as very low for all oral health outcomes. We have very little confidence in the effect estimate and acknowledge that the true effect is likely to be substantially different from the estimate of effect. Studies were downgraded because of limitations in allocation concealment, lack of intention to treat analysis and blinding of participants, those delivering treatment and outcome assessors. In addition, interventions were delivered differently in different settings and some did not have an adequate sample size. Details of certainty of evidence assessment are available in Table 4.

**Table 4:** Summary of findings

Certainty assessment							No. of patients		Effect		Certainty	Importance
No. of studies	Study design	Risk of bias	Inconsistency	Indirectness	Imprecision	Other considerations	Experiment	Control	Relative (95% CI)	Absolute (95% CI)		
*Dental caries measured by DMFT scores*												
7	4 Randomised trials 2 Quasi experiments 1 Non-randomized trial	Serious^a^	Not serious	Serious^b^	Serious^c^	None	4392	2374	–	SMD − **0.33** (− 0.56, − 0.10)	⨁◯◯◯ Very low	IMPORTANT
*Dental caries measured by net increment in DMFT scores*												
5	3 Randomised trials 2 non-randomized trial	Serious^a^	Not serious	Serious^b^	Serious^d^	None	3091	2401	–	SMD − **0.34** (− 0.69, 0.02)	⨁◯◯◯ Very low	IMPORTANT
*Dental caries measured by DMFS scores*												
5	3 Randomised trials 1 quasi experiment 1 cohort study	Serious^e^	Not serious	Serious^b^	Serious^f^	None	2110	1396	–	SMD − **0.26** (− 0.70, 0.18)	⨁◯◯◯ Very low	IMPORTANT
*Dental caries measured by net increment in DMFS scores*												
3	2 Randomised trials 1 Cohort study	Serious^e^	Not serious	Serious^b^	Serious^g^	None	2066	1689	–	SMD − **1.09** (− 1.91, 0.27)	⨁◯◯◯ Very low	IMPORTANT
*Dental caries prevalence measured by dmft or DMFT/S score greater than one*												
4	1 Randomised trial 1 Quasi experiment 2 cohort studie	Serious^a^	Not serious	Serious^b^	Serious	None	871/2049 (42.5%)	553/919 (60.2%)	**RR 0.70** (0.53, 0.94)	–	⨁◯◯◯ Very low	IMPORTANT
*Oral hygiene measured by plaque scores*												
7	All randomised trials	Serious^a^	Not serious	Serious^b^	Serious^i^	None	1971	1368	–	SMD − **0.32** (− 0.46, − 0.18)	⨁◯◯◯ Very low	IMPORTANT
*Oral hygiene measured by gingival scores*												
3	All randomised trials	Serious^a^	Not serious	Serious^b^	Serious	None	1094	479	–	SMD **0.12** (− 0.32, 0.55)	⨁◯◯◯ Very low	IMPORTANT

The bold provides information on certainty of assessement, number of pateints, effect, certainty and importance

*CI* confidence interval, *RR* risk ratio, *SMD* standardized mean difference

^a^Studies show limitations for allocation concealment, blinding of participants, those delivering treatment and outcome assessors, and intention to treat analysis

^b^Interventions delivered differently in different settings

^c^Five of seven studies did not have the required sample (200) in each group to provide optimal information size (OIS)

^d^Two out of five studies did not have the required sample (200) in each group to provide optimal information size (OIS)

^e^Studies show limitations with allocation concealment; blinding of participants, those delivering treatment and outcome assessors; intention to treat analysis and control confounding

^f^Four out of five studies did not have the required sample (200) in each group to provide optimal information size (OIS)

^g^One out of three studies did not have the required sample (200) in each group to provide optimal information size (OIS)

^h^Three out of four studies did not have the required sample (200) in each group to provide optimal information size (OIS)

^i^Six out of seven studies did not have the required sample (200) in each group to provide optimal information size (OIS)

^j^All three studies did not have the required sample (200) in each group to provide optimal information size (OIS)

## Discussion

To our knowledge, this systematic review is the first to assess effectiveness of school-based interventions in improving oral health of primary school children in low- and middle-income countries. Critically, the review showed that interventions incorporating skills-based education into oral health education programs had a significant positive effect on reduction of plaque and dental caries scores.

In addition, studies that incorporated teacher training, provision of health services, engaging parents and changing school environment had positive effect on oral health outcomes.

Previous trials have shown that oral hygiene instructions and/or information to students on the causes and prevention of oral diseases, facilitated a rise in oral health awareness [29, 31, 32, 34, 40, 44, 46, 49], potentially improving healthy behaviours and oral health outcomes. A cross-sectional study that examined oral health status and possible risk factors in China found children with higher oral health knowledge scores were less likely to have dental caries and gingival bleeding [54]. The WHO recommends skills-based education using teaching and learning methods commensurate with available resources to prevent oral diseases among school children. Hence, such skills-based education should be incorporated as strategies in school-based interventions to manage oral diseases.

We also found that studies that incorporated teacher training into oral health education programs showed a significant positive effect on dental caries [8, 27, 34, 52], plaque [31, 49], gingival health [8, 27, 42], oral health practices [8, 32, 34, 36, 46], attitude [34, 46], quality of life [42], beliefs [42], and sense of coherence [42]. Interventions included training teachers on content and delivery of oral health education, which could have resulted in children acquiring health knowledge and skills and a subsequent reduction in oral diseases. While teachers are important in the implementation of school oral health education, their lack of knowledge on causes and prevention of oral diseases and delivery of oral health education has been documented [55, 56]. For school-based interventions to be beneficial it is important to train teachers on content and delivery of oral health education programs. This promotes sustainability while building understanding, skills and attitudes to enable teachers to deliver oral health education competently and confidently.

Our meta-analysis including three studies indicated that providing access to school health services reduced dental caries [9, 34, 52] and plaques scores [9, 34]. In these trials, children were screened and provided treatment for oral diseases, which could have helped in prevention and control of oral diseases and encouraged healthy behaviour. Access to care is an important determinant of oral health and yet children in many LMICs have poor access to oral health care [57]. Dependent on available resources and oral health status of children, schools can adopt an appropriate model of providing oral health services to benefit children.

Studies that engaged parents by providing oral health education sessions, involving parents in delivery of interventions to children or providing reports on oral health status, also showed a positive effect for oral health outcomes [8, 9, 27, 28, 30, 32, 33, 43, 46, 48, 53]. This could have reinforced health promotion activities at home and influenced behaviour towards prevention of oral diseases, thereby producing positive effects on oral health outcomes. A review on evidence of the influence of parents’ oral health behaviours on their children’s dental caries reported that parent’s oral hygiene knowledge, attitude and practices were related to children’s oral health status and behaviour [58]. Parental involvement in oral health education programs should be considered when developing school-based interventions to provide children with adequate oral health.

Changes to the school environment could have contributed to providing a supportive environment that encouraged healthy lifestyles and behaviours conducive to oral health. A cross sectional study that assessed the relationship between social environment and oral health related quality of life found that children in healthier environments reported fewer oral symptoms, functional limitation, and better social well-being [59]. Providing a healthy environment could be important in adopting healthy lifestyles and behaviours but more studies are required to evaluate this aspect in-depth.

Duration of interventions appears to be important, as shown by findings of our subgroup analyses that dental caries were significantly lower in children who received interventions that lasted two years or more compared to interventions that lasted less than two years. We are not able to make comparison of sensitivity and subgroup analyses with a previous review [12] as these were not conducted because of insufficient number of studies.

It should be noted that the certainty of evidence was assessed as very low for all oral health outcomes due to several methodologic and design factors. Previous reviews report similar findings [12–14]. As such, though our final finding needs to be interpreted with caution, it also highlights the urgent need for high quality dental research in this area.

Our review provides insight into the impact of primary school-based interventions on oral health related outcomes among schoolchildren. The provision of skills-based education in improving oral health outcomes, knowledge, attitude, behaviour and quality of life cannot be overemphasised. The choice of teaching and learning methods would depend on the resources in the school. Using trained teachers to deliver the intervention was effective. In addition, providing access to oral health services such as oral examination, fissure sealants, screening and treatment to reduce dental caries and plaques scores, and improve oral health practices, gingival health and knowledge seem to be effective. Parents could be engaged in reinforcing health promotion activities at home and influencing behaviour towards prevention of oral diseases. Interventions could consider longer periods for effectiveness on dental caries outcomes.

## Limitations

Even though we conducted an extensive search strategy, we limited the publications to English language only. Therefore, the effectiveness of the interventions could be overrepresented. Only one author completed the initial title screen to exclude articles which were obviously not relevant to the review. It is therefore possible that we may have missed some eligible studies during screening. However, we hand searched reference lists from eligible trials and relevant systematic reviews to identify any potentially relevant trials to reduce our chances of missing eligible studies.

The studies in our review had limitations with weaknesses in the quality of their methodologies. These may have weakened the results of the studies included in our review. Most studies had limitations for allocation concealment, blinding of participants, those delivering treatment and outcome assessors, and intention to treat analysis. However, these were unavoidable because of the nature of the intervention. In addition, we were not able to formally assess publication bias and cannot rule it out.

## Conclusion

Based on our meta-analysis, school-based interventions can be effective in reducing the burden of oral disease among primary school children in LMICs. Based on the narrative synthesis, incorporating skills-based education with teacher training, providing access to oral health services, engaging parents and changing the school environment is crucial in preventing oral diseases among schoolchildren.

### Implications for practice

Our review provides evidence that school-based interventions can be effective at improving several oral health outcomes in schoolchildren. It appears that duration and therefore sustainability, of interventions is important for success. Based on current evidence we are unable to determine the impact of school-based interventions on other than oral health outcomes such as fruit and vegetable intake, socialisation, school attendance, and academic performance.

Oral health is part of general health and affects quality of life. The WHO provides a comprehensive framework [17] that includes the interventions that we have found to be effective. LMICs can adopt intervention models from this framework, dependent on resources, to control oral diseases among primary school children.

### Implications for research

There is an urgent need for more high-quality research to assess the effectiveness of school-based interventions on oral health. Specifically, more research should seek to determine whether improving health promoting policies is effective in addressing oral health outcomes. More evaluations should seek to determine whether oral health outcomes are best addressed by longer intervention durations.

Interventions should be guided by theory and provide clear descriptions of interventions. Future experimental studies should clearly describe methods of randomisation of participants, allocation concealment, blinding of participants, blinding persons delivering the intervention and outcome assessors, intention to treat analysis, statistical power analysis and trial design. Future observational studies should recruit participants free of the outcome of interest at the start of the study and clearly describe strategies to address incomplete follow up and confounding.

## Supplementary Information

**Additional file 1.** Preferred reporting items for systematic reviews and meta-analyses (PRISMA) checklist. A 27-item checklist addressing title, abstract, introduction, methods, results, discussion and funding sections of a systematic review report. For reference, page numbers of each section are indicated.

**Additional file 2.** Studies excluded with reasons for exclusion. The file provides a list of 71 references of studies that were excluded from the review and reasons for exclusion of each study.

**Additional file 3.** Characteristics of included studies. The document provides characteristics of included studies with information on: WHO region, author(s) and year of publication, country, participants age, intervention design, outcomes measured and study design.

**Additional file 4.** Studies with positive effect on oral health outcomes. The document provides characteristics of studies with positive effect on oral health outcomes including author(s) and year of publication, study design, intervention design, duration of intervention and effect of intervention compared to controls.

**Additional file 5.** Sensitivity analysis to examine the influence on the summary effect estimates or all meta-analyses. The file provides a list of studies excluded for each sensitivity analysis, reasons for exclusion, summary effects when studies have been included and when both random effects and fixed effect models are used. In addition, summary effects are provided for use of both relative risks and odds ratios for one meta-analysis.

**Additional file 6.** Search strategy for Ovid MEDLINE (May 2020).

## Data Availability

All data generated or analysed during this study are included in this published article and its additional files.

## Abbreviations

dmft/DMFT: Decayed, missing and filled teeth
dmfs/DMFS: Decayed, missing and filled tooth surfaces
GRADE: Grading of recommendations, assessment, development and evaluation
JBI: Joanna Briggs Institute
LMICs: Low- and middle-income countries
NCD: Non-communicable disease
PRISMA: Preferred reporting items for systematic reviews and meta-analyses
RR: Risk ratio
SoF: Summary of findings
UNSW: University of New South Wales
WHO: World Health Organisation
