# Supplementary material for: Effectiveness of primary school-based interventions in improving oral health of children in low- and middle-income countries: a systematic review and meta-analysis
Source: BMC Oral Health. 2022 Jun 29;22:264. doi: 10.1186/s12903-022-02291-2 (PMC9245251; doi:10.1186/s12903-022-02291-2)
Supplement: Supplementary file 2 — Additional file 2. Studies excluded with reasons for exclusion. The file provides a list of 71 references of studies that were excluded from the review and reasons for exclusion of each study. [file 12903_2022_2291_MOESM2_ESM.docx]

**Studies excluded with reasons for exclusion.**

1. Ahmad M. Effectiveness of Health Education to Improve Oral Care of Primary School Children in a Rural Community of Pakistan. EC Dental Science. 2019; 18:01-9.

**Reasons for exclusion:** No comparator.

1. Borges-Yanez SA, Castrejon-Perez RC, Camacho MEI. Effect of a School-Based Supervised Tooth Brushing Program In Mexico City: A Cluster Randomized Intervention. Journal of Clinical Pediatric Dentistry. 2017;41(3):204-13.

**Reasons for exclusion:** No comparator.

1. Alsumait A, ElSalhy M, Behzadi S, Raine KD, Gokiert R, Cor K, et al. Impact evaluation of a school-based oral health program: Kuwait National Program. BMC Oral Health. 2019;19(1):202.

**Reasons for exclusion:** Wrong setting, the study was conducted in Kuwait.

1. Amini R, Vanaki V. The survey of the effectiveness of oral health game on the dental health status of the school children of Isfahan. 2003.

**Reasons for exclusion:** Wrong language.

1. Angelopoulou MV, Kavvadia K, Taoufik K, Oulis CJ. Comparative clinical study testing the effectiveness of school based oral health education using experiential learning or traditional lecturing in 10-year-old children BMC Oral Health 2015;15(1):1-7.

**Reasons for exclusion:** Wrong setting. The study was conducted in Greece.

1. Angelopoulou MV, Oulis CJ, Kavvadia K. School-based oral health-education program using experiential learning or traditional lecturing in adolescents: a clinical trial. International Dental Journal. 2014;64(5):278-84.

**Reasons for exclusion:** Wrong setting. The study was conducted in Greece.

1. Baca P, Munoz MJ, Bravo M, Junco P, Baca AP. Effectiveness of chlorhexidine-thymol varnish for caries reduction in permanent first molars of 6-7-year-old children: 24-month clinical trial. Community Dentistry & Oral Epidemiology. 2002;30(5):363-8.

**Reasons for exclusion:** Wrong setting. The study was conducted in Spain

1. Bardeskar S, Prasad S, Luke F, Doss K. A study to assess the effectiveness of planned health teaching programme on knowledge regarding oral hygiene practices among children (8–10 years) in a selected school at Rajkot, Gujarat. International Journal of Advances in Nursing Management. 2018;6(2):98-100.

**Reasons for exclusion:** No comparator.

1. Blake H, Dawett B, Leighton P, Rose-Brady L, Deery C. School-Based Educational Intervention to Improve Children's Oral Health-Related Knowledge. Health Promotion Practice. 2015;16(4):571-82.

**Reasons for exclusion:** Wrong setting, the study was conducted in United Kingdom (UK).

1. Bordoni N, Bellagamba H, Dono R, Piovano S, Marcantoni M, Squassi A. Effect of self-brushing with acidulated phosphate fluoride (pH 5.6) on dental caries in children. Acta Odontologica Latinoamericana. 1994;8(2):17-25.

**Reasons for exclusion:** Wrong timing, the study was published in 1994

1. Brambilla E, Gagliani M, Felloni A, Garcia-Godoy F, Strohmenger L. Caries-preventive effect of topical amine fluoride in children with high and low salivary levels of mutans streptococci. Caries Research. 1999;33(6):423-7.

**Reasons for exclusion:** Wrong setting, the study was conducted in Italy

1. Bravo Perez M, Llodra Calvo JC, Baca Garcia P, Osorio Ruiz E, Junco Lafuente P. [Fissure sealants versus fluorine varnish on the first permanent molars: economic assessment]. Atencion Primaria. 1995;15(3):143-7.

**Reasons for exclusion:** Wrong setting, the study was conducted in Spain.

1. Calderon Larranaga S, Exposito Ruiz M, Cruz Vela P, Cuadrado Conde A, Alquezar Villarroya L, Garach Gomez A, et al. Primary Care and oral health promotion: Assessment of an educational intervention in school children. Atencion Primaria. 2019;51(7):416-23.

**Reasons for exclusion:** Wrong setting, the study was conducted in Spain.

1. Carpino R, Walker MP, Liu Y, Simmer-Beck M. Assessing the effectiveness of a school-based dental clinic on the oral health of children who lack access to dental care: a program evaluation. The Journal of School Nursing. 2017;33(3):181-8.

**Reasons for exclusion:** Wrong setting, the study was conducted in United Sates of America (USA)

1. Chapman A, Copestake S, Duncan K. An oral health education programme based on the National Curriculum. International journal of paediatric dentistry. 2006;16(1):40-4.

**Reasons for exclusion:** Wrong setting. The study was conducted in England

1. Clark E, Foster Page L, Larkins K, Leon de la Barra S, Thomson WM. Caries-preventive efficacy of a supervised school toothbrushing programme in Northland, New Zealand. Community Dent Health. 2019.

**Reasons for exclusion:** Wrong setting. The study was conducted in New Zealand

1. Cueto AU, Barraza AS, Munoz DA, Chang S. Evaluation of an Oral Health Promotion and Preventive Programme: A Case-Control Study. Oral Health & Preventive Dentistry. 2016;14(1):49-54.

**Reasons for exclusion:** Wrong population. The study population comprised clinical records

1. Curnow MM, Pine CM, Burnside G, Nicholson JA, Chesters RK, Huntington E. A randomised controlled trial of the efficacy of supervised toothbrushing in high-caries-risk children. Caries Research. 2002;36(4):294-300.

**Reasons for exclusion:** Wrong setting. The study was conducted in Scotland.

1. D'Cruz A, Aradhya S. Impact of oral health education on oral hygiene knowledge, practices, plaque control and gingival health of 13- to 15-year-old school children in Bangalore city. International Journal of Dental Hygiene. 2013;11(2):126-33.

**Reasons for exclusion:** Wrong population. The study was among 13-15-year-old children. There is no mention if it was a Primary School or Secondary school.

1. Divaris K, Rozier RG, King RS. Effectiveness of a School-based Fluoride Mouthrinse Program. Journal of Dental Research. 2011;91(3):282-7.

**Reasons for exclusion:** Wrong setting. The study was conducted in USA

1. Donaldson M, Kinirons M. Effectiveness of the school dental screening programme in stimulating dental attendance for children in need of treatment in Northern Ireland. Community Dentistry & Oral Epidemiology. 2001;29(2):143-9.

**Reasons for exclusion:** Wrong setting. The study was conducted in N. Ireland.

1. Drosen C, Goddon I, Heinrich-Weltzien R. [Evaluation of a school-based intensified preventive program for high caries risk children in the Eneppe-Ruhr district]. Bundesgesundheitsblatt, Gesundheitsforschung, Gesundheitsschutz. 2010;53(11):1197-204.

**Reasons for exclusion:** Wrong setting. The study was conducted in Germany.

1. Folayan MO, Sofola OO, Oginni AB. Caries incidence in a cohort of primary school students in Lagos State, Nigeria followed up over a 3 years period. European Archives of Paediatric Dentistry: Official Journal of the European Academy of Paediatric Dentistry. 2012;13(6):312-8.

**Reasons for exclusion:** No comparator.

1. Freeman R, Oliver M. Do school break-time policies influence child dental health and snacking behaviours? An evaluation of a primary school programme. British Dental Journal. 2009;206(12):619-25; discussion 6. **Reasons for exclusion:** Wrong setting. The study was conducted in Ireland
2. Friel S, Hope A, Kelleher C, Comer S, Sadlier D. Impact evaluation of an oral health intervention amongst primary school children in Ireland. Health Promotion International. 2002;17(2):119-26.

**Reasons for exclusion:** Wrong setting. The study was conducted in Ireland.

1. Gasoyan H, Safaryan A, Sahakyan L, Gasoyan N, Aaronson WE, Bagramian RA. School-Based Preventive Dental Program in Rural Communities of the Republic of Armenia. Frontiers in Public Health. 2019;7. **Reasons for exclusion:** No comparator
2. Gauba A, Bal IS, Jain A, Mittal HC. School based oral health promotional intervention: Effect on knowledge, practices and clinical oral health related parameters. Contemporary clinical dentistry. 2013;4(4):493-9. **Reasons for exclusion:** No comparator
3. Goel P, Sehgal M, Mittal R. Evaluating the effectiveness of school-based dental health education program among children of different socioeconomic groups. Journal of Indian Society of Pedodontics and Preventive Dentistry. 2005;23(3):131-3.

**Reasons for exclusion:** No comparator

1. Gold J. Fluoride Varnish With Community-Based Oral Health Promotion May Reduce Surface-Level Caries Risk in Preschool Children. Journal of Evidence-Based Dental Practice. 2013;13(2):55-7.

**Reasons for exclusion:** Wrong setting. The study was conducted in USA.

1. Halawany HS, Al Badr A, Al Sadhan S, Al Balkhi M, Al-Maflehi N, Abraham NB, et al. Effectiveness of oral health education intervention among female primary school children in Riyadh, Saudi Arabia. Saudi Dental Journal. 2018;30(3):190-6.

**Reasons for exclusion:** Wrong setting. The study was conducted in Saudi Arabia.

1. Halonen H, Pesonen P, Seppä L, Peltonen E, Tjäderhane L, Anttonen V. Outcome of a Community-Based Oral Health Promotion Project on Primary Schoolchildren’s Oral Hygiene Habits. International Journal of Dentistry. 2013;2013:485741.

**Reasons for exclusion:** Wrong setting. The study was conducted in Finland.

1. Haque SE, Rahman M, Itsuko K, Mutahara M, Kayako S, Tsutsumi A, et al. Effect of a school-based oral health education in preventing untreated dental caries and increasing knowledge, attitude, and practices among adolescents in Bangladesh. BMC Oral Health. 2016;16(1):44. **Reasons for exclusion:** Wrong population: Study among high school students
2. Hardman MC, Davies GM, Duxbury JT, Davies RM. A cluster randomised controlled trial to evaluate the effectiveness of fluoride varnish as a public health measure to reduce caries in children. Caries Research. 2007;41(5):371-6. **Reasons for exclusion:** Wrong setting. The study was conducted in England.
3. Harikiran AG, Vadavi D, Shruti T. Beta Testing an Oral Health Edutainment Card Game Among 12-13 Year Old Children in Bangalore, India. Games for Health Journal. 2017;6(6):334-42.

**Reasons for exclusion:** No comparator

1. Hariyani N, Setyowati D, Aristyanti N, Setijanto D. Natural peer group approach as a learning strategist for maximizing dental health education in school-age children. J Int Oral Health. 2020;12(1):27-32**.**

**Reasons for exclusion:** No comparator

1. Iwakura ML, Morita MC. [Fluoride mouth-rinsing to prevent dental caries in a Brazilian municipality with fluoridated drinking water]. Pan American Journal of Public Health. 2004;15(4):256-61.

**Reasons for exclusion:** Wrong language

1. Jackson RJ, Newman HN, Smart GJ, Stokes E, Hogan JI, Brown C, et al. The effects of a supervised toothbrushing programme on the caries increment of primary school children, initially aged 5-6 years. Caries Research. 2005;39(2):108-15.

**Reasons for exclusion:** Wrong setting. The study was conducted in England.

1. Josie-Perez A, Chikte UM, Rudolph MJ, Bellingan A, Brand AA. The impact of a mobile dental system on a school community. Part I--Oral health status of schoolchildren. Journal of the Dental Association of South Africa. 1994;49(10):491-4. **Reasons for exclusion:** The study was published in 1994
2. Lalloo R, Solanki GS. An evaluation of a school-based comprehensive public oral health care programme. Community Dental Health. 1994;11(3):152-5. **Reasons for exclusion:** The study was published in 1994
3. Lalloo R, Kroon J, Tut O, Kularatna S, Jamieson LM, Wallace V, et al. Effectiveness, cost-effectiveness and cost-benefit of a single annual professional intervention for the prevention of childhood dental caries in a remote rural Indigenous community. BMC Oral Health. 2015;15(1):99. **Reasons for exclusion:** Wrong setting. The study was conducted in Australia.
4. Lambert MJ, De Visschere LMJ, Martens LC, Deschepper E, Vanobbergen J. The impact of a prospective 4‐year longitudinal school intervention for improving oral health and oral health inequalities in primary schoolchildren in Flanders‐Belgium. International Journal of Paediatric Dentistry. 2019;29(4):439–47.

**Reasons for exclusion:** Wrong setting. The study was conducted in Belgium.

1. Louw AJ, Carstens IL, Hartshorne JE, Blignaut RJ. Effectiveness of two school-based caries preventive programmes. Journal of the Dental Association of South Africa. 1995;50(2):43-9. **Reasons for exclusion:** No comparator. Baseline examination provided data against which experimental cohorts were annually compared.
2. Luis HS, Morgado I, Assuncao V, Bernardo MF, Leroux B, Martin MD, et al. Dental hygiene work in a clinical trial. International journal of dental hygiene. 2008;6(3):238-43.

**Reasons for exclusion:** Wrong setting. The study was conducted in Portugal.

1. Martin AB, Hardin JW, Veschusio C, Kirby HA. Differences in dental service utilization by rural children with and without participation in head start. Pediatric Dentistry. 2012;34(5):e107-e11. **Reasons for exclusion:** Wrong setting. The study was conducted in USA.
2. Masood M, Yusof N, Hassan MI, Jaafar N. Assessment of dental caries predictors in 6-year-old school children - results from 5-year retrospective cohort study. BMC Public Health. 2012;12:989. **Reasons for exclusion:** No comparator
3. Masood M, Yusof N, Hassan MI, Jaafar N. Longitudinal study of dental caries increment in Malaysian school children: a 5-year cohort study. Asia-Pacific Journal of Public Health. 2014;26(3):260-7.**Reasons for exclusion:** No comparator
4. Mazzocchi AR, Moretti R. Effectiveness of a dental preventive program on plaque index results in 13-year-old children of Bergamo, Italy. Community Dentistry and Oral Epidemiology. 1997;25(4):332-3. **Reasons for exclusion:** Wrong setting. The study was conducted in Italy.
5. Milsom K, Blinkhorn A, Worthington H, Threlfall A, Buchanan K, Kearney-Mitchell P, et al. The effectiveness of school dental screening: a cluster-randomized control trial. Journal of Dental Research. 2006;85(10):924-8. **Reasons for exclusion**: Wrong setting. The study was conducted in England.
6. Milsom KM, Threlfall AG, Blinkhorn AS, Kearney-Mitchell PI, Buchanan KM, Tickle M. The effectiveness of school dental screening: dental attendance and treatment of those screened positive. British Dental Journal. 2006;200(12):687-90; discussion 73. **Reasons for exclusion**: Wrong setting. The study was conducted in England.
7. Milsom KM, Blinkhorn AS, Walsh T, Worthington HV, Kearney-Mitchell P, Whitehead H, et al. A cluster-randomized controlled trial: fluoride varnish in school children. Journal of Dental Research. 2011;90(11):1306-11. **Reasons for exclusion**: Wrong setting. The study was conducted in England.
8. Moskovitz M, Abud W, Ram D. The Influence of an Oral Health Education Program Provided in a Community Dental Clinic on the Prevalence of Caries Among 12-14 Year-Old Children. Journal of Clinical Pediatric Dentistry. 2009;33(3):259-64. **Reasons for exclusion**: Wrong setting. The study was conducted in Israel.
9. Moysés ST, Moysés SJ, Watt RG, Sheiham A. Associations between health promoting schools’ policies and indicators of oral health in Brazil. Health Promotion International. 2003;18(3):209-18. **Reasons for exclusion**: No comparator.
10. Najari N, Mehdizadeh AH, Mehdizadeh M, Nodoushan ZJ, Mohammadbeigi. A. A Comparison between Conventional and Video Methods of Health Education on Improvement of Oral Health in Elementary School Students of Qomrood Village, 2018, (Iran). . Qom University of Medical Sciences Journal. 2019;13(5):25-32. **Reasons for exclusion:** Wrong language. Abstract in English, full text in Arabic
11. Petersen PE, Hunsrisakhun J, Thearmontree A, Pithpornchaiyakul S, Hintao J, Jurgensen N, et al. School-based intervention for improving the oral health of children in southern Thailand. Community Dental Health. 2015;32(1):44-50. **Reasons for exclusion:** Wrong population. The study involved pre-school children
12. Pieper K, Weber K, Margraf-Stiksrud J, Stein S, Heinzel-Gutenbrunner M, Jablonski-Momeni A. Evaluation of an intensified preventive programme aimed at 12-year-olds with increased caries risk. Journal of Public Health (Germany). 2012;20(2):151-7. **Reasons for exclusion:** Wrong setting. The study was conducted in Germany.
13. Poornima P, Sathyaki Arora NI, Shashibhushan K, Nagaveni N. An Effectiveness of Oral Health Promotion Program on 12 Year old School Going Children of South Indian Children. **Reasons for exclusion:** No full text
14. Qadri G, Alkilzy M, Franze M, Hoffmann W, Splieth C. School-based oral health education increases caries inequalities. Community Dental Health. 2018;35(3):153-9. **Reasons for exclusion:** Wrong setting. The study was conducted in Germany.
15. Redmond CA, Blinkhorn FA, Kay EJ, Davies RM, Worthington HV, Blinkhorn AS. A cluster randomized controlled trial testing the effectiveness of a school-based dental health education program for adolescents. Journal of Public Health Dentistry. 1999;59(1):12-7. **Reasons for exclusion:** Wrong setting. The study was conducted in England.
16. Reinhardt CH, Lopker N, Noack MJ, Klein K, Rosen E. Peer Tutoring Pilot Program for the Improvement of Oral Health Behavior in Underprivileged and Immigrant Children. Pediatric Dentistry. 2009;31(7):481-5. **Reasons for exclusion:** Wrong setting. The study was conducted in Germany.
17. Rodrigues JA, Dos Santos PA, Baseggio W, Milori Corona SA, Palma-Dibb RG, Sasso Garcia PPN. Oral hygiene indirect instruction and periodic reinforcements: Effects on index plaque in schoolchildren. Journal of Clinical Pediatric Dentistry. 2009;34(1):31-4. **Reasons for** **exclusion:** No comparator
18. Ruff RR, Niederman R. Comparative effectiveness of school-based caries prevention: a prospective cohort study. BMC Oral Health. 2018;18(1):53. **Reasons for exclusion:** Wrong setting. The study was conducted in USA.
19. Saied-Moallemi Z. Oral Health among Iranian Preadolescents: A School-Based Health Education Intervention. University of Helsinki: University of Helsinki; 2010. **Reasons for exclusion:** Duplicate publication. Thesis that has manuscript published.
20. Saied-Moallemi Z, Murtomaa H, Virtanen JI. Change in conceptions of iranian pre-adolescents' oral health after a school-based programme: Challenge for boys. Oral Hearlth Prev Dent. 2014;12(1):21-8. **Reasons for exclusion:** No full text
21. Shahraki-Sanavi F, Ansari-Moghaddam A, Rakhshani F, Mohammadi M. Effectiveness of school-based education programs on health risk behaviors in adolescents: South-east of Iran. Iranian Red Crescent Medical Journal. 2018;20(Supplement 2):e62034. **Reasons for exclusion:** Wrong population: Study was conducted among adolescents in public high schools.
22. Swe KK. Effectiveness of oral health education on primary school children and oral health awareness and practice of caregivers. University of Public Health Yangon: University of Public Health Yangon; 2017.  **Reasons for exclusion**: Duplicate publication.
23. olvanen M, Lahti S, Poutanen R, Seppa L, Pohjola V, Hausen H. Changes in children's oral health-related behavior, knowledge and attitudes during a 3.4-yr randomized clinical trial and oral health-promotion program. European Journal of Oral Sciences. 2009;117(4):390-7. **Reasons for exclusion**: Wrong setting. The study was conducted in Finland.
24. Vanobbergen J, Declerck D, Mwalili S, Martens L. The effectiveness of a 6-year oral health education programme for primary schoolchildren. Community dentistry and oral epidemiology. 2004;32(3):173-82. **Reasons for exclusion**: Wrong setting. The study was conducted in Denmark.
25. Weitz A, Marinanco MI, Villa A. Reduction of caries in rural school-children exposed to fluoride through a milk-fluoridation programme in Araucania, Chile. Community Dental Health. 2007;24(3):186-91. **Reasons for exclusion**: Wrong setting. The study was conducted in Chile.
26. Wolff MS, Hill R, Wilson-Genderson M, Hirsch S, Dasanayake AP. Nationwide 2.5-Year School-Based Public Health Intervention Program Designed to Reduce the Incidence of Caries in Children of Grenada. Caries Research. 2016;50 Suppl 1:68-77.  **Reasons for exclusion:** No comparator.
27. Worthington HV, Hill KB, Mooney J, Hamilton FA, Blinkhorn AS. A cluster randomized controlled trial of a dental health education program for 10-year-old children. Journal of Public Health Dentistry. 2001;61(1):22-7. **Reasons for exclusion:** Wrong setting. The study was conducted in England.
28. Zhang SY, Dong H, Yu M. [The effects of different interventions on 12-year-old children's permanent teeth caries and filling rate in Shanghai Jiading district]. Shanghai Kou Qiang Yi Xue/Shanghai Journal of Stomatology. 2015;24(3):341-4. **Reasons for exclusion:** Wrong language. Chinese
