# Supplementary material for: Effectiveness of primary school-based interventions in improving oral health of children in low- and middle-income countries: a systematic review and meta-analysis
Source: BMC Oral Health. 2022 Jun 29;22:264. doi: 10.1186/s12903-022-02291-2 (PMC9245251; doi:10.1186/s12903-022-02291-2)
Supplement: Supplementary file 5 — Additional file 5. Sensitivity analysis to examine the influence on the summary effect estimates or all meta-analyses. The file provides a list of studies excluded for each sensitivity analysis, reasons for exclusion, summary effects when studies have been included and when both random effects and fixed effect models are used. In addition, summary effects are provided for use of both relative risks and odds ratios for one meta-analysis. [file 12903_2022_2291_MOESM5_ESM.docx]

Additional file 5: Sensitivity analysis to examine the influence on the summary effect estimates or all meta-analyses.

| **Sensitivity analysis** | **Studies dropped because of poor quality** | | |
| --- | --- | --- | --- |
|  | **Excluded studies** | **Reasons for exclusion** | **Summary effect estimates** |
| **Sensitivity analysis of effect of school-based intervention on dental caries experience by measurement of DMFT scores.** | 1. Pakhomov *et al*, 1997 (39)  2. Duijster *et al*, 2017 (23) | Selection bias | Standardised Mean Difference (SMD) = -0.19 (95%CI -0.43, 0.04; P = 0.10) |
|  | 1. Naidu & Nandlal, 2017 (34)  2. Hartono *et al*, 2002 (31) | Detection bias | SMD = -0.44 (95%CI -0.71, -0.17; P = 0.002) |
|  | 1. Naidu & Nandlal, 2017 (34)  2. Pakhomov *et al*, 1997 (39)  3. Van Wyk *et al*, 2004 (25) | Attrition bias | SMD = -0.03 (95%CI -0.10, 0.03; P = 0.32) |
|  | 1. Hartono *et al*, 2002 (31)  2. Naidu & Nandlal, 2017 (34)  3. Pakhomov *et al*, 1997 (39)  4.Van Palenstein et al, 1997 (36)  5. Van Wyk *et al*, 2004 (25) | Small sample size (Less than 200 in each arm) | SMD = -0.03 (95%CI -0.10, 0.04; P = 0.46) |
|  | **Use of both random effects and fixed effect models.** | | |
|  | **Model** | **Summary effect estimates** | |
|  | Random effects model | SMD = -0.33 (95%CI - 0.56, -0.10; P = 0.005) | |
|  | Fixed effect model. | SMD = -0.22 (95%CI -0.27, -0.17; P < 0.00001) | |
| **2. Sensitivity analysis of effect of school-based intervention on dental caries experience by measurement net increment in DMFT scores.** | **Studies dropped because of poor quality** | | |
|  | **Excluded studies** | **Reasons for exclusion** | **Summary effect estimates** |
|  | 1. Duijster *et al*, 2017 (23)  2. Jamie *et al*, 2015 (32) | Selection bias. | SMD = -0.39 (95%CI -0.89, 0.10; P = 0.12) |
|  | 1. Naidu & Nandlal, 2017 (34) | Detection bias | SMD = -0.34 95%CI -0.79, 0.11; p= 0.14) |
|  | 1. Naidu & Nandlal, 2017 (34) | Attrition bias | SMD = -0.34 95%CI -0.79, 0.11; p= 0.14). |
|  | 1. Naidu & Nandlal, 2017 (34)  2. Jamie *et al*, 2015 (32) | Small sample size (Less than 200 in each arm) | SMD = -0.36 (95%CI -0.88, 0.16; P = 0.17). |
|  | **Use of both random effects and fixed effect models.** | | |
|  | **Model** | **Summary effect estimates** | |
|  | Random effects model | SMD = -0.34 (95%CI -0.69, 0.02; P = 0.06) | |
|  | Fixed effect model. | SMD = -0.38 (95%CI -0.44, -0.33; P < 0.00001) | |
| **3. Sensitivity analysis of effect of school-based intervention on dental caries experience by measurement of DMFS scores.** | **Studies dropped because of poor quality** | | |
|  | **Excluded studies** | **Reasons for exclusion** | **Summary effect estimates** |
|  | 1. Naidu & Nandlal, 2017 (34)  2. Monse *et al*, 2013 (24)  3. Hartono *et al*, 2002 (31)  4. Frencken *et al,* 2001 (37) | Small sample size (Less than 200 in each arm) | SMD = -0.05 95%CI -0.16, 0.05; p = 0.29). |
|  | **Use of both random effects and fixed effect models.** | | |
|  | **Model** | **Summary effect estimates** | |
|  | Random effects model | SMD = -0.26; 95%CI -0.70 to 0.18; P = 0.24) | |
|  | Fixed effect model. | SMD = -0.17 (95%CI -0.25, -0.10; P < 0.00001). | |
| **4: Sensitivity and group analysis of the effect of school-based intervention on dental caries experience by measurement of net increment in DMFS scores.** | **Studies dropped because of poor quality** | | |
|  | **Excluded studies** | **Reasons for exclusion** | **Summary effect estimates** |
|  | 1. Monse *et al*, 2013 (24)  2. Peng *et al*, 2004 (27)  3. Naidu & Nandlal, 2017 (34) | Selection bias. | SMD = -1.53 95%CI -1.65, -1.41; P < 0.00001) |
|  | 1. Monse *et al*, 2013 (24)  2. Peng *et al*, 2004 (27)  3. Naidu & Nandlal, 2017 (34) | Detection bias | SMD = -1.53 95%CI -1.65, -1.41; P < 0.00001) |
|  | 1. Naidu & Nandlal, 2017 (34) | Attrition bias | SMD = -0.73 95%CI -1.54, 0.07; P = 0.08) |
|  | 1. Naidu & Nandlal, 2017 (34)  2. Monse *et al*, 2013 (24) | Small sample size (Less than 200 in each arm) | SMD = -0.57 95%CI -1.56, 0.42; P = 0.26). |
|  | **Use of both random effects and fixed effect models.** | | |
|  | **Model** | **Summary effect estimates** | |
|  | Random effects model | SMD = -1.09 (95%CI -1.91, -0.27; P = 0.009) | |
|  | Fixed effect model. | SMD = -0.85 (95%CI -0.92, -0.78; P < 0.00001). | |
| **5. Sensitivity analysis of the effect of school-based intervention on dental caries prevalence by measurement of dmft or DMFT/S score greater than one.** | **Studies dropped because of poor quality** | | |
|  | **Excluded studies** | **Reasons for exclusion** | **Summary effect estimates** |
|  | 1. De sousa *et al*, 2002 (41)  2. Lai *et al*, 2016 (42) | Cohort study | RR 0.85 (95%CI 0.59, 1.23; P = 0.39) |
|  | **Use of both random effects and fixed effect models.** | | |
|  | **Model** | **Summary effect estimates** | |
|  | Random effects model | Relative risk (RR) = 0.70 (95%CI 0.53, 0.94; P = 0.02  Odds ratio (OR) = 0.43 (95%CI 0.21, 0.87: P = 0.02) | |
|  | Fixed effect model. | RR = 0.68 (95%CI 0.63, 0.74; P < 0.00001)  OR = 0.44 (95%CI 0.37, 0.52: P < 0.00001) | |
| **6. Sensitivity and subgroup analysis of the effect of school-based intervention on oral hygiene by measurement of plaque scores.** | **Studies dropped because of poor quality** | | |
|  | **Excluded studies** | **Reasons for exclusion** | **Summary effect estimates** |
|  | 1. Frencken *et al,* 2001 (37)  2. Hartono *et al*, 2002 (31)  3. Naidu & Nandlal, 2017 (34)  4. Simpriano & Mialhe, 2017 (47) | Detection bias | SMD = -0.31 [-0.50, -0.12; P = 0.001). |
|  | 1. Chauhan *et al*, 2016 (30)  2. Hebbal *et a*l, 2011 (44)  3. Naidu & Nandlal, 2017 (34) | Attrition bias | SMD = -0.26 (95%CI -0.43, -0.09; P = 0.03). |
|  | 1. Nyandeni *et al*, 1996 (49) | Small sample size (Less than 200 in each arm) | SMD = -0.35 (95%CI -0.51, -0.19; P < 0.00001) |
|  | **Use of both random effects and fixed effect models.** | | |
|  | **Model** | **Summary effect estimates** | |
|  | Random effects model | SMD= -0.32; 95% CI: -0.46, -0.18; P < 0.00001) | |
|  | Fixed effect model. | SMD = -0.30 (95%CI -0.37, -0.22; P < 0.00001) | |
| **7. Sensitivity and subgroup analysis of the effect of school-based intervention on oral hygiene by measurement of mean gingival scores.** | **Studies dropped because of poor quality** | | |
|  | **Excluded studies** | **Reasons for exclusion** | **Summary effect estimates** |
|  | 1. Naidu & Nandlal, 2017 (34)  2. Simpriano & Mialhe, 2017 (47) | Detection bias | SMD = 0.50 (95%CI 0.30, 0.70; P < 0.00001). |
|  | 1. Naidu & Nandlal, 2017 (34)  2. Simpriano & Mialhe, 2017 (47) | Performance bias | SMD = 0.50 (95%CI 0.30, 0.70; P < 0.00001). |
|  | 1. Naidu & Nandlal, 2017 (34)  2. Chauhan *et al*, 2016 (30) | Attrition bias | SMD = -0.11 (95%CI -0.36, 0.14; P = 0.40) |
|  | **Use of both random effects and fixed effect models.** | | |
|  | **Model** | **Summary effect estimates** | |
|  | Random effects model | SMD = 0.12 (95%CI -0.32, 0.55; P = 0.60) | |
|  | Fixed effect model. | SMD = -0.08 (95%CI -0.20, 0.04; P = 0.21) | |
