# Supplementary material for: Effectiveness of primary school-based interventions in improving oral health of children in low- and middle-income countries: a systematic review and meta-analysis
Source: BMC Oral Health. 2022 Jun 29;22:264. doi: 10.1186/s12903-022-02291-2 (PMC9245251; doi:10.1186/s12903-022-02291-2)
Supplement: Supplementary file 6 — Additional file 6. Search strategy for Ovid MEDLINE (May 2020). [file 12903_2022_2291_MOESM6_ESM.docx]

**Appendix 1: Search strategy for Ovid MEDLINE. Searched on 27/05/2020**

1. "oral health".mp. [mp=title, abstract, original title, name of substance word, subject heading word, floating sub-heading word, keyword heading word, organism supplementary concept word, protocol supplementary concept word, rare disease supplementary concept word, unique identifier, synonyms

2. "dental health".mp. [mp=title, abstract, original title, name of substance word, subject heading word, floating sub-heading word, keyword heading word, organism supplementary concept word, protocol supplementary concept word, rare disease supplementary concept word, unique identifier, synonyms]

3. School.mp. [mp=title, abstract, original title, name of substance word, subject heading word, floating sub-heading word, keyword heading word, organism supplementary concept word, protocol supplementary concept word, rare disease supplementary concept word, unique identifier, synonyms]

4. “oral health promotion".mp. [mp=title, abstract, original title, name of substance word, subject heading word, floating sub-heading word, keyword heading word, organism supplementary concept word, protocol supplementary concept word, rare disease supplementary concept word, unique identifier, synonyms]

5. "oral health education".mp. [mp=title, abstract, original title, name of substance word, subject heading word, floating sub-heading word, keyword heading word, organism supplementary concept word, protocol supplementary concept word, rare disease supplementary concept word, unique identifier, synonyms]

6. children.mp. [mp=title, abstract, original title, name of substance word, subject heading word, floating sub-heading word, keyword heading word, organism supplementary concept word, protocol supplementary concept word, rare disease supplementary concept word, unique identifier, synonyms]

7. child.mp. [mp=title, abstract, original title, name of substance word, subject heading word, floating sub-heading word, keyword heading word, organism supplementary concept word, protocol supplementary concept word, rare disease supplementary concept word, unique identifier, synonyms]

8. intervention.mp. [mp=title, abstract, original title, name of substance word, subject heading word, floating sub-heading word, keyword heading word, organism supplementary concept word, protocol supplementary concept word, rare disease supplementary concept word, unique identifier, synonyms]

9. effectiveness.mp. [mp=title, abstract, original title, name of substance word, subject heading word, floating sub-heading word, keyword heading word, organism supplementary concept word, protocol supplementary concept word, rare disease supplementary concept word, unique identifier, synonyms]

10. "randomized controlled trial".mp. [mp=title, abstract, original title, name of substance word, subject heading word, floating sub-heading word, keyword heading word, organism supplementary concept word, protocol supplementary concept word, rare disease supplementary concept word, unique identifier, synonyms]

11. "before and after study".mp. [mp=title, abstract, original title, name of substance word, subject heading word, floating sub-heading word, keyword heading word, organism supplementary concept word, protocol supplementary concept word, rare disease supplementary concept word, unique identifier, synonyms]

12. "pre and post study".mp. [mp=title, abstract, original title, name of substance word, subject heading word, floating sub-heading word, keyword heading word, organism supplementary concept word, protocol supplementary concept word, rare disease supplementary concept word, unique identifier, synonyms]

13. pre-post study".mp. [mp=title, abstract, original title, name of substance word, subject heading word, floating sub-heading word, keyword heading word, organism supplementary concept word, protocol supplementary concept word, rare disease supplementary concept word, unique identifier, synonyms]

14. "interrupted time-series".mp. [mp=title, abstract, original title, name of substance word, subject heading word, floating sub-heading word, keyword heading word, organism supplementary concept word, protocol supplementary concept word, rare disease supplementary concept word, unique identifier, synonyms]

15. "case control study".mp. [mp=title, abstract, original title, name of substance word, subject heading word, floating sub-heading word, keyword heading word, organism supplementary concept word, protocol supplementary concept word, rare disease supplementary concept word, unique identifier, synonyms]

16. "cohort study".mp. [mp=title, abstract, original title, name of substance word, subject heading word, floating sub-heading word, keyword heading word, organism supplementary concept word, protocol supplementary concept word, rare disease supplementary concept word, unique identifier, synonyms]

17. "cross-sectional study".mp. [mp=title, abstract, original title, name of substance word, subject heading word, floating sub-heading word, keyword heading word, organism supplementary concept word, protocol supplementary concept word, rare disease supplementary concept word, unique identifier, synonyms]

18. Oral Health/

19. Dental Care/ or Dental Caries/ or Health Education, Dental/ or Oral Health/

20. Schools/

21. Dental Care/ or Oral Health/ or Oral Hygiene/ or Health Promotion/ or Health Education, Dental/

22. Oral Hygiene/ or Health Knowledge, Attitudes, Practice/ or Oral Health/ or Dental Care/ or Health Education, Dental/

23. Child/

24. Child Health Services/ or Child Health/ or Child/

25. Early Medical Intervention/ or Early Intervention, Educational/ or Internet-Based Intervention/

26. Comparative Effectiveness Research/

27. Randomized Controlled Trial/

28. Cohort Studies/

29. Case-Control Studies/

30. Cross-Sectional Studies/

31. 1 or 2 or 18 or 19

32. 3 or 20

33. 4 or 5 or 21 or 22

34. 6 or 7 or 23 or 24

35. 8 or 9 or 25 or 26

36. 10 or 11 or 12 or 13 or 14 or 15 or 16 or 17 or 27 or 28 or 29 or 30

37. 31 and 32 and 33 and 34 and 35 and 36
